# Protoilludene and Alkenoic Acid Derivatives from the European Polypore *Fomitiporia hartigii*

**DOI:** 10.1021/acsomega.4c04287

**Published:** 2024-07-04

**Authors:** Winnie Chemutai Sum, Sherif S. Ebada, Hao Wang, Harald Kellner, Marc Stadler

**Affiliations:** † Department of Microbial Drugs, Helmholtz Centre for Infection Research GmbH (HZI), Inhoffenstraße 7, 38124 Braunschweig, Germany; ‡ Institute of Microbiology, Technische Universität Braunschweig, Spielmannstraße 7, 38106 Braunschweig, Germany; § Department of Pharmacognosy, Faculty of Pharmacy, Ain Shams University, 11566 Cairo, Egypt; ∥ Key Laboratory of Natural Products Research and Development of Li Folk Medicine of Hainan Province, 1 Institute of Tropical Bioscience and Biotechnology, Chinese Academy of Tropical Agricultural Sciences, Haikou, Hainan 571101, China; ⊥ Department of Bio- and Environmental Sciences, Technische Universität Dresden-International Institute Zittau, Markt 23, 02763 Zittau, Germany

## Abstract

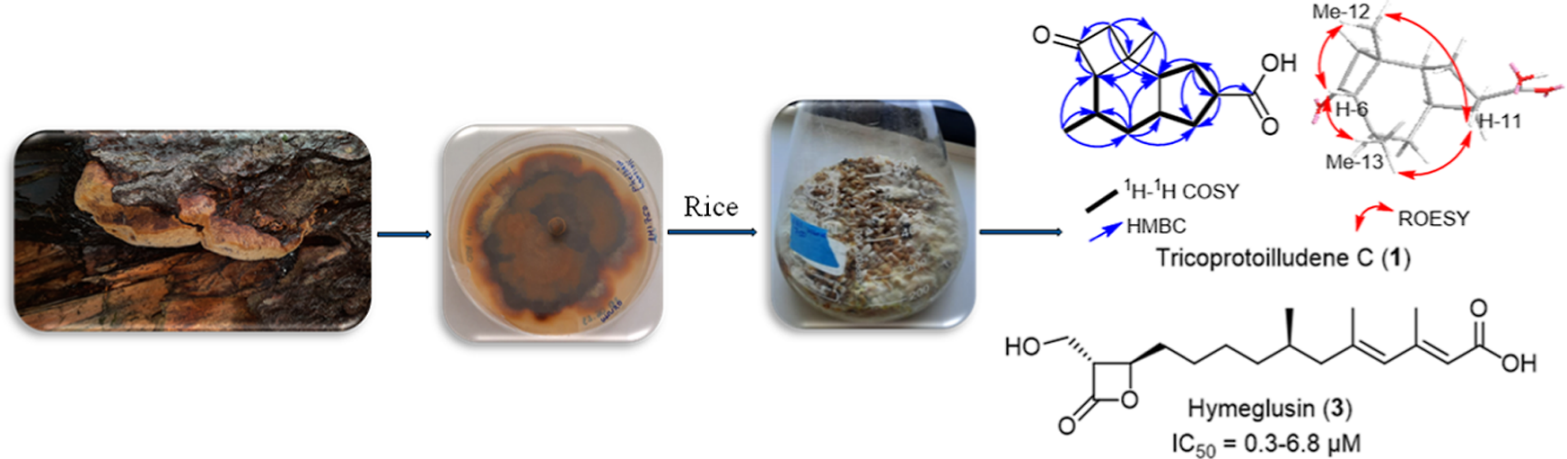

Chemical investigation of the solid-state rice culture of the endangered European polypore *Fomitiporia hartigii* (Hymenochaetaceae) afforded a previously undescribed protoilludene derivative (**1**) in addition to six known compounds (**2**–**7**). Chemical structures of the isolated compounds were established based on HR-ESI-MS, comprehensive 1D/2D NMR spectroscopic analyses, and comparisons with the literature. All isolated compounds were assessed for their cytotoxic and antimicrobial activities. Among the tested compounds, hymeglusin (**3**) revealed potent cytotoxic activity against all tested cell lines with IC_50_ values between 0.3 and 6.8 μM. Compound **3** and fusaridioic acid A (**4**) revealed weak to moderate antimicrobial activities with its most potent effect against *Candida albicans* (minimum inhibitory concentration of 4.2 μg/mL).

## Introduction

With more than 40,000 species worldwide, Basidiomycota represents the second largest phylum of Kingdom Fungi.^1^ Over the years, basidiomycetes have introduced a considerable number of bioactive secondary metabolites with significant bioactivities such as antimicrobial, antiproliferative, antihyperlipidemic, hepato-, and neuroprotective.^1,2^ Our study fungus *Fomitiporia hartigii* (often-synonymized *Phellinus hartigii*) belongs to the fungal family Hymenochaetaceae, currently composed of thirty-four accepted genera based on morphological and molecular characterization.^3^ It is noteworthy that fungi of genera *Fomitiporia*, *Fuscoporia*, *Fulvifomes*, *Tropicoporus*, *Porodaedalea*, *Ochrosporellus*, *Sanghuangporus*, *Flaviporellus* Murill, *Mensularia*, and *Onnia*, among others, were initially considered synonymic to *Phellinus sensu lato* or *Inonotus sensu lato*. Nonetheless, further phylogenetic studies revealed the genera monophyletic and independent,^3,4^ hence moved to distinct genera.

Most species of the family Hymenochaetaceae are forest pathogens, with considerable economic significance. Some species have been widely studied for their interesting therapeutic properties, e.g., *Inonotus obliquus* (“Chaga”)^5^ and *Sanghuangporus sanghuang* (“Sanghuang”).^6^ Nevertheless, the genus *Fomitiporia* has not been studied extensively for its secondary metabolites. Despite that, most species of the genus have been implicated as causal agents for the Esca disease of grapevines.^7,8^ Our previous exploration of the Kenyan *Fomitiporia aethiopica* led to the discovery of previously unprecedented steroids with moderate cytotoxic activities.^9^ Recently, we encountered *F. hartigii*, an endangered European species known to be closely associated with fir (*Abies* spp.). Thus, inspired by our previous success with the related *F. aethiopica*, we chemically explored the axenic solid-state rice culture of *F. hartigii*. Herein, we report the results obtained from the chemical and pharmacological prospections.

## Results and Discussion

### Strain Identification

The fungal specimen (IHI 750) was collected and morphologically identified by one of the authors (H.K.). The specimen was found on a recently fallen *Abies alba* log of decay class 1 at Mittelsteighütte in the Bavarian Forest National Park in Bavaria, Germany, in May 2022. This location is known since the 1960s to harbor this German red listed fungus (i.e., category: near threatened).^10^ A copy of the mycelial culture of the fungus was deposited at the German Collection of Microorganisms and Cell Cultures (DSMZ), Braunschweig, Germany, under no. STMA 23102.

### Structure Elucidation of Secondary Metabolites

**Figure 1:**
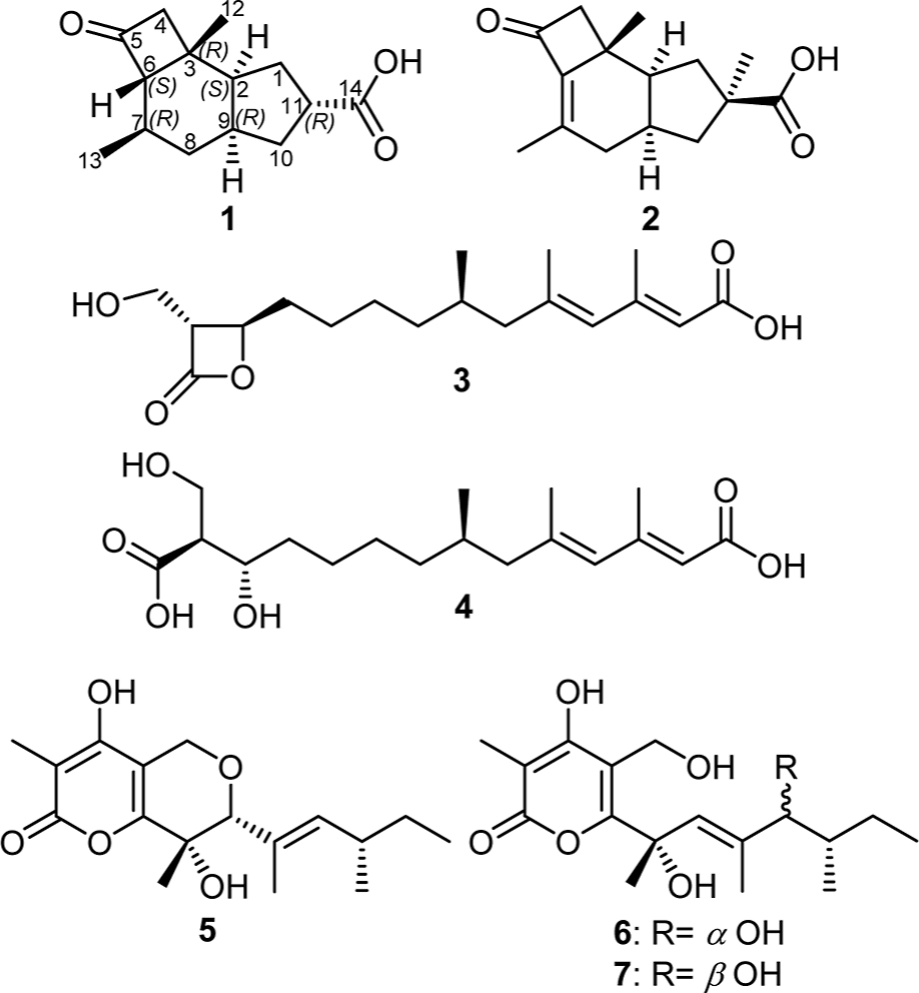
Chemical structures of **1**–**7**.

Dereplication study of EtOAc extract derived from the solid-state rice culture of the European polypore *F. hartigii* was conducted implementing HPLC–DAD–MS supported by secondary metabolite database searches (https://dnp.chemnetbase.com/chemical/ChemicalSearch.xhtml?dswid=-621), and it revealed the presence of one previously undescribed protoilludene derivative and six known compounds (Figure 1). Chemical structures of the isolated compounds were elucidated, based on HR-ESI-MS, 1D/2D NMR spectroscopic analyses and the comparisons with the reported literature. The known compounds were determined as tricoprotoilludene A (**2**),^11^ hymeglusin (**3**),^12^ fusaridioic acid A (**4**),^12^ neovasinin (**5**),^13^ and neovasiopyrones A (**6**) and B (**7**).^14^ All of the isolated compounds were assessed for their cytotoxic and antimicrobial activities against a panel of cell lines and microbes, respectively.

**Figure 2:**
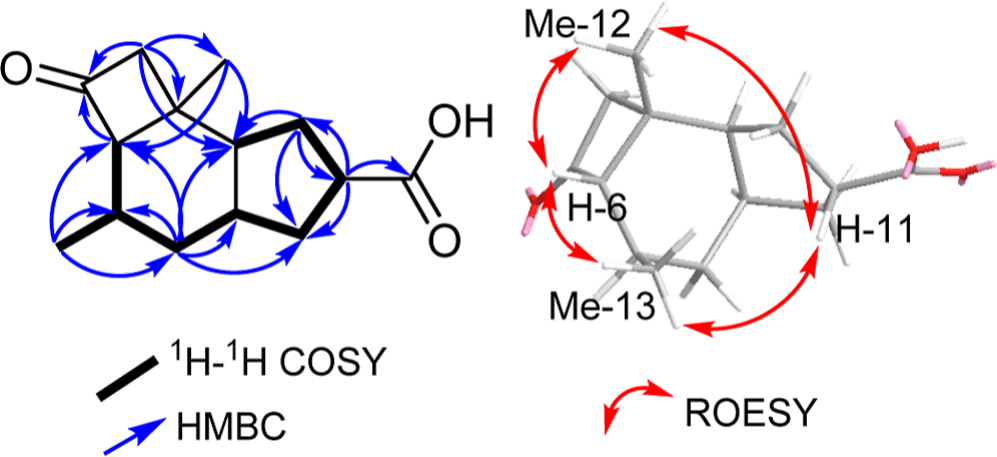
Key ^1^H– ^1^H COSY, HMBC, and ROESY correlations of **1**.

Compound **1** was purified as a white amorphous inseparable solid mixture with tricoprotoilludene A (**2**). HR-ESI-MS established its molecular formula as C_14_H_20_O_3_ indicating five degrees of unsaturation by revealing a protonated molecular ion peak at *m*/*z* 237.1480 [M + H]^+^ (calculated 237.1485) and a sodium adduct at *m*/*z* 259.1303 [M + Na]^+^ (calculated 259.1305). The ^13^C NMR spectral data of **1** (Table 1) revealed the presence of two carbonyl carbon atoms differentiated into one ketocarbonyl carbon (δ_C_ 211.4) and one carboxylic acid carbon (δ_C_ 182.0) accounting for two degrees of unsaturation and thus suggesting a tricyclic sesquiterpenoidal structure for **1**. In addition, the ^13^C NMR and HSQC spectra of **1** (Table 1, Figures S7, and S10) revealed the presence of 12 sp^3^ carbon atoms recognized into one unprotonated (δ_C_ 31.8), five methines (δ_C_ 69.4, 44.9, 42.1, 40.5, and 32.2), four methylenes (δ_C_ 57.5, 37.9, 35.0, and 31.7), and two methyl groups (δ_C_ 27.5 and 22.3). A literature search of **1** based on the obtained results suggested it to comprise a protoilludene sesquiterpenoidal skeleton closely related to tricoprotoilludenes and compounds that were given the trivial names “phellinharts A–C”, recently reported from two basidiomycetous fungi *Daedaleopsis tricolor*(7) and “*P. hartigii”*,^15^ respectively. A detailed comparison of both ^1^H and ^13^C NMR data of **1** to those reported for tricoprotoilludenes and phellinharts revealed its close resemblance to “phellinhart C^15”^ except for the absence of the methyl ester group. In addition, we compared the solvents used during our extraction, isolation, and purification schemes to those adopted to attain phellinharts. In our scheme where we avoided any exposure to methanol, only the free carboxylic acid form of phellinhart C was purified and identified as compound **1**, whereas the scheme followed by Zheng et al. involved several column chromatographic separations using mobile phases including methanol as a solvent and they purified phellinhart C as a methyl ester together with another sesquiterpene lactone methyl ester, phellinhart A.^15^ These notions might suggest that phellinharts A and C as methyl esters perhaps emerged from an esterification reaction that took place during chromatographic workups. To affirm or negate this assumption, a backward tracing of phellinharts A and C to the crude extract of their fungal source might be requested. Further confirmation of the depicted structure of **1** (Figure 1) was obtained by the HMBC spectrum (Figures 2 and S9) that revealed key correlations from a diastereotopic methylene protons at δ_H_ 2.45/3.22 (H_2_-4) to a ketocarbonyl carbon at δ_C_ 211.4 (C-5) and a methyl carbon at δ_H_ 27.5 (C-12). In addition, key correlations have also been revealed from two methyl groups (H_3_-12 and H_3_-13) to a methine carbon at δ_H_ 32.2 (C-7) and from a proton signal at δ_H_ 2.92 (dddd, *J* = 10.8, 9.2, 7.4, 3.5 Hz, H-11) to a terminal carboxylic acid carbon at δ_C_ 182.0 (C-14). The relative configuration of **1** was determined by the ROESY spectrum (Figure 2) that revealed key ROE correlations between two methyl groups H_3_-12/H_3_-13 and two methine protons H-6/H-11 indicating four of them to be directed toward the same face of the molecule while H-2, H-7, and H-9 are directed toward the opposite side of the molecule. By comparing the ^1^H and ^13^C NMR spectral data of **1** (Table 1) and “phellinhart C^15”^ whose absolute configuration was determined by X-ray crystallography and taking in consideration their common biosynthetic origin, the absolute configuration of **1** was conclusively determined as (2*S*,3*R*,6*S*,7*R*,9*R*,11*R*). The obtained results confirmed that compound **1** is a previously undescribed protoilludene derivative that was trivially named tricoprotoilludene C.

**Table 1:** 1D (^1^H and ^13^C) NMR Data of **1**

pos.	δ_C_,^a^ type	δ_H_^a^ multi (***J*** [Hz])
1	31.7, CH_2_	α 1.81 overlapped
		β 2.05 overlapped
2	44.9, CH	2.19 overlapped
3	31.8, C	
4	57.5, CH_2_	α 2.45 dd (16.0, 6.1)
		β 3.22 dd (16.0, 1.4)
5	211.4, CO	
6	69.4, CH	2.18 overlapped
7	32.2, CH	1.64 dtt (13.1, 6.6, 3.4)
8	35.0, CH_2_	α 0.86 q (12.7)
		β 1.40 dt (13.1, 3.9)
9	40.5, CH	2.24 overlapped
10	37.9, CH_2_	α 1.82 overlapped
		β 2.09 overlapped
11	42.1, CH	2.92 dddd (10.8, 9.2, 7.4, 3.5)
12	27.5, CH_3_	1.21 d (0.8)
13	22.3, CH_3_	1.01 d (6.6)
14	182.0, CO	

a Measured in methanol-*d*_4_ at 125 MHz for ^13^C and 500 MHz for ^1^H.

### Biological Activity of Compounds **1**–**7**

Using our standardized protocols,^16−18^ all isolated compounds were subjected to cytotoxic and antimicrobial activity assays against two cell lines, namely, mouse fibroblasts (L929) and human endocervical adenocarcinoma (KB3.1) whereas the antimicrobial activity was determined against 12 different bacterial and fungal pathogens (Table 2). For compounds featuring potent cytotoxic activity, a further assessment against five additional cell lines was conducted.

**Table 2:** Cytotoxicity (IC_50_) and Antimicrobial Activity [Minimum Inhibitory Concentration (MIC)] of **1**–**7**^a^

	IC_50_ (μM)					positive control
test cell line	**1/2**	**3**	**4**	**5**	**6/7**	Epothilone B (nM)
mouse fibroblast (L929)	n.a.	6.8	n.a.	n.a.	n.a.	0.65
human endocervical adenocarcinoma (KB3.1)	n.a.	2.2	n.a.	n.a.	n.a.	0.17
human prostate carcinoma (PC-3)	n.d.	0.3	n.d.	n.d.	n.d.	0.09
human breast adenocarcinoma (MCF-7)	n.d.	4.0	n.d.	n.d.	n.d.	0.07
human ovarian cancer (SKOV-3)	n.d.	2.2	n.d.	n.d.	n.d.	0.09
human epidermoid carcinoma (A431)	n.d.	2.6	n.d.	n.d.	n.d.	0.06
human lung carcinoma (A549)	n.d.	n.a.	n.d.	n.d.	n.d.	0.05

test microorganism	MIC (μg/mL)					positive control (μg/mL)
*Staphylococcus aureus*	n.i.	66.6	66.6	n.i.	n.i.	0.42^G^
*Escherichia coli*	n.i.	n.i.	n.i.	n.i.	n.i.	0.83^G^
*Bacillus subtilis*	n.i.	n.i.	66.6	n.i.	n.i.	16.6°
*Pseudomonas aeruginosa*	n.i.	n.i.	n.i.	n.i.	n.i.	0.42^G^
*Pichia anomala*	n.i.	33.3	n.i.	n.i.	n.i.	8.30^N^
*Candida albicans*	n.i.	4.2	n.i.	n.i.	n.i.	8.30^N^
*Acinetobacter baumannii*	n.i.	n.i.	n.i.	n.i.	n.i.	1.04^C^
*Chromobacterium violaceum*	n.i.	n.i.	n.i.	n.i.	n.i.	1.67^G^
*Schizosaccharomyces pombe*	n.i.	66.6	66.6	n.i.	n.i.	8.30^N^
*Mucor hiemalis*	n.i.	66.6	n.i.	n.i.	n.i.	8.30^N^
*Rhodotorula glutinis*	n.i.	66.6	n.i.	n.i.	n.i.	4.20^N^
*Mycobacterium smegmatis*	n.i.	n.i.	n.i.	n.i.	n.i.	1.70^K^

a n.a.: no activity. n.i.: no inhibition up to 67 μg/mL. n.d.: not determined. G: gentamicin; O: oxytetracycline; N: nystatin; C: ciprofloxacin; K: kanamycin.

The obtained results (Table 2) revealed that among the tested compounds, hymeglusin (**3**) induced significant cytotoxic activity against all tested cell lines except human lung carcinoma (A549) with IC_50_ values ranging from 0.3 to 6.8 μM. In the antimicrobial assay (Table 2), hymeglusin (**3**) also revealed potent antifungal activity against *C. albicans* at MIC of 4.2 μg/mL whereas it revealed together with fusaridioic acid A (**4**) weak antimicrobial activities against few bacterial and fungal strains with MIC values of 33.3–66.6 μg/mL (Table 2).

## Conclusions

In this study, an EtOAc crude organic extract of the European polypore *F. hartigii* was chemically explored, implementing a dereplication scheme followed by chromatographic workup to purify the potentially new natural products. Results unveiled the presence of one previously undescribed protoilludene derivative, tricoprotoilludene C (**1**) in addition to six known metabolites (**2**–**7**). In the cytotoxic and antimicrobial activity assays, hymeglusin (**3**) revealed potent cytotoxic activity against most of the tested cell lines and antifungal activity against *C. albicans* that could be attributed to the *β*-lactone functionality in its structure which is opened in fusaridioic acid A (**4**) lacking activity in these assays. This study valorizes rare Basidiomycota and fungi in general as a prolific source of bioactive natural products that could be of potential applications.

## Materials and Methods

### General Experimental Procedures

HPLC–DAD/MS samples were analyzed on an amaZon speed ETD ion trap mass spectrometer (Bruker Daltonics, Bremen, Germany) in positive and negative ionization modes. The HPLC system consisted of a Dionex UltiMate 3000 UHPLC (Thermo Fisher Scientific Inc., Waltham, MA, USA), equipped with a C_18_ Acquity UPLC BEH column (Waters, Milford, USA). The mobile phase consisted of solvent A [deionized water +0.1% formic acid (FA)] and solvent B [acetonitrile (MeCN) + 0.1% FA]. The gradient used was as follows: 5% B for 0.5 min, 100% B over a period of 20 min, and then holding the 100% B for 10 min, at a flow rate of 0.6 mL/min. UV–vis detections were obtained at 190–600 and 210 nm wavelengths. HR-(+)ESI-MS measurements were performed in the positive ionization mode using the timsTOF Pro 2 mass spectrometer (Bruker Daltonics, Bremen, Germany) connected to an Agilent 1290 series HPLC-UV system (Agilent Technologies, Santa Clara, CA, USA) equipped with a C_18_ Acquity UPLC BEH column (Waters, Milford, USA). The used eluent were solvents A (deionized water + 0.1% FA) and B (MeCN +0.1% FA). The gradient of separation was as follows: 5% B for 0.5 min, 100% B over a period of 20 min, and keeping the 100% B for 5 min. The flow rate was 0.6 mL/min (40 °C) and UV–vis detections were made at 200–600 nm. Molecular formulas of the secondary metabolites were obtained using the Smart Formula algorithm of Compass DataAnalysis software (Bruker Daltonics, version 6.1). NMR spectra of compounds (dissolved in methanol-*d*_4_) were recorded on an AVANCE III 500 (Bruker, ^1^H: 500 MHz, ^13^C: 125 MHz) spectrometer (Daltonics, Bremen, Germany). Optical rotation was determined using an Anton Paar MCP-150 polarimeter (Graz, Austria). The UV spectra were recorded on a Shimadzu UV-2450 UV–vis spectrophotometer (Kyoto, Japan) at a concentration of 0.02 mg/mL in methanol. Solvents (analytical and HPLC grade) and chemicals were purchased from AppliChem GmbH (Darmstadt, Germany), Carl Roth GmbH & Co., KG (Karlsruhe, Germany), Avantor Performance Materials (Deventer, Netherlands), and Merck (Darmstadt, Germany). Deionized water was obtained from the PURELAB flex water purification system (Veolia Water Technologies, Celle, Germany).

### Fungal Specimen and Preparation of Cultures

A fruiting body of *F. hartigii* (IHI 750) was collected, and pieces of the pore region of the underside were cut and placed on water agar. After a successful growth, the culture was transferred on malt agar plates. The isolate generally showed a slow growing behavior.

### Fermentation of Cultures and Extraction of Metabolites

YMG (4 g of yeast extract, 10 g of malt extract, 4 g of glucose, and 20 g of agar in 1 L deionized water) medium plates were prepared and used to inoculate 6 × 500 mL Erlenmeyer culture flasks, containing sterile rice medium as previously described.^14^ Subsequently, secondary metabolites were extracted after static incubation at 24 °C in the dark for 28 days to yield a crude extract (1.2 g) as initially reported.^14^

### Isolation of Compounds and Their Physico-Chemical Properties

The aforementioned EtOAc extract (1.2 g) was purified on a preparative HPLC system (PLC 2020; Gilson, Middleton, WI, USA). The solvent system consisted of deionized water + 0.1% FA (solvent A) and MeCN + 0.1% FA (solvent B) using a VP NUCLEODUR 100–5C_18_ column (250 × 40 mm, 7 μm: Macherey-Nagel, Düren, Germany).

The gradient implemented for elution of the compounds was as follows: an initial isocratic start condition at 5% solvent B for 10 min, followed by an increase from 5 to 25% of solvent B within 3 min, thereafter another increase from 25 to 65% solvent B in 120 min, and a final holding of the gradient at 100% solvent B for 10 min. The flow rate was 40 mL/min and UV detections were made at 190, 210, and 280 nm wavelengths. The separation yielded **6** and **7** (2.58 mg, *t*_R_ = 31 min), **4** (9.73 mg, *t*_R_ = 47 min), **1** and **2** (11.45 mg, *t*_R_ = 57–59 min), **5** (5.48 mg, *t*_R_ = 61 min), and **3** (13.40 mg, *t*_R_ = 70–72 min).

#### Tricoprotoilludenes C (**1**) and A (**2**)

Pink oil; [α]_D_^20^ −13° (*c* 0.1, methanol); UV–vis (MeOH): λ_max_ = 257, 210 nm; NMR data (^1^H NMR: 500 MHz, ^13^C NMR: 125 MHz, methanol-*d*_4_), see Table 1; HR-(+)ESI-MS for **1**: *m*/*z* 219.1375 [M – H_2_O + H]^+^ (calcd 219.1380 for C_14_H_19_O_2_^+^), 237.1480 [M + H]^+^ (calcd 237.1485 for C_14_H_21_O_3_^+^), 259.1303 [M + Na]^+^ (calcd 259.1305 for C_14_H_20_NaO_3_^+^); HR-(+)ESI-MS for **2**: *m*/*z* 231.1376 [M – H_2_O + H]^+^ (calcd 231.1380 for C_15_H_19_O_2_^+^), 249.1484 [M + H]^+^ (calcd 249.1485 for C_15_H_21_O_3_^+^), 271.1304 [M + Na]^+^ (calcd 271.1305 for C_15_H_20_NaO_3_^+^); *t*_R_ = 7.28 min (LR-ESI-MS).

### Antimicrobial Assay

Antimicrobial activity of the isolated secondary metabolites was determined using our established protocol,^16−18^ using clinically relevant microorganisms obtained from the German Collection of Microorganisms and Cell Cultures (DSMZ, Braunschweig, Germany). These microorganisms included *S. aureus* (DSM 346), *B. subtilis* (DSM 10), *A. baumannii* (DSM 30008), *E. coli* (DSM 1116), *C. violaceum* (DSM 30191), *P. aeruginosa* (PA14), *M. smegmatis* (ATCC 700084), *C. albicans* (DSM 1665), *M. hiemalis* (DSM 2656), *R. glutinis* (DSM 10134), *S. pombe* (DSM 70572), and *P. anomala* (DSM 6766). The MIC was recorded in each case as the lowest concentration at which no microbial growth was visualized.

### Cytotoxicity (MTT) Assay

In vitro cytotoxicity of the purified compounds was established based on the MTT [3-(4,5-dimethylthiayol-2-yl)-2,5-diphenyltetrazolium bromide] assay as previously reported.^16−18^ The mammalian cell lines used in the tests were sourced from DSMZ and included mouse fibroblasts (L929), human lung carcinoma (A549), endocervical adenocarcinoma (KB3.1), prostate carcinoma (PC-3), breast adenocarcinoma (MCF-7), and epidermoid carcinoma cells (A431).

## Supporting Information Available

HPLC–ESI-MS, HR-ESI-MS, 1D (^1^H/^13^C), and 2D (^1^H–^1^H COSY, HMBC, HSQC, and ROESY) NMR spectra of compounds (**1**–**7**) (PDF)
