# Supplementary material for: Protoilludene and Alkenoic Acid Derivatives from the European Polypore Fomitiporia hartigii
Source: ACS Omega. 2024 Jul 4;9(28):31006–10. doi: 10.1021/acsomega.4c04287 (PMC11256317; doi:10.1021/acsomega.4c04287)
Supplement: Supplementary file 1 — ao4c04287_si_001.pdf [file ao4c04287_si_001.pdf]

Supporting Information for:

Protoilludene and Alkenoic Acid Derivatives from the European Polypore *Fomitiporia hartigii*

Winnie Chemutai Sum,[†,‡] Sherif S. Ebada,*[†,§] Hao Wang,[⊥] Harald Kellner,[¶] and Marc Stadler*[†, ‡]

[†] Department of Microbial Drugs, Helmholtz Centre for Infection Research GmbH (HZI), Inhoffenstraße 7, 38124 Braunschweig, Germany.

[‡] Institute of Microbiology, Technische Universität Braunschweig, Spielmannstraße 7, 38106 Braunschweig, Germany.

[§] Department of Pharmacognosy, Faculty of Pharmacy, Ain Shams University, 11566 Cairo, Egypt.

[⊥] Key Laboratory of Natural Products Research and Development of Li Folk Medicine of Hainan Province, Institute of Tropical Bioscience and Biotechnology, Chinese Academy of Tropical Agricultural Sciences, Haikou, Hainan 571101, China.

[¶] Department of Bio- and Environmental Sciences, Technische Universität Dresden-International Institute Zittau, Markt 23, 02763 Zittau, Germany.

* Correspondence: sherif.elsayed@helmholtz-hzi.de; sherif_elsayed@pharma.asu.edu.eg (S.S.E.); Marc.Stadler@helmholtz-hzi.de (M.S.); Tel.: +49-531-6181-4240; Fax +49-531-6181-9499

Contents of Supporting Information

#	Contents	Page
**1**	Figure S1. Key ^{1}H–^{1}H COSY, HMBC and ROESY correlations of **2**.	**S3**
**2**	Table S1. ^{1}H and ^{13}C NMR data of compound (**2**) and tricoprotoilludene A.^{a}	**S3**
**3**	Figure S2. LR-ESI-MS of **1**.	**S4**
**4**	Figure S3. LR-ESI-MS of **2**.	**S5**
**5**	Figure S4. HR-ESI-MS of **1**.	**S6**
**6**	Figure S5. HR-ESI-MS of **2**.	**S7**
**7**	Figure S6. ^{1}H NMR spectrum of **1** and **2** in methanol-d_4 at 500 MHz.	**S8**
**8**	Figure S7. ^{13}C NMR spectrum of **1** and **2** in methanol-d_4 at 125 MHz.	**S9**
**9**	Figure S8. ^{1}H-^{1}H COSY spectrum of **1** and **2** in methanol-d_4 at 500 MHz.	**S10**
**10**	Figure S9. HMBC spectrum of **1** and **2** in methanol-d_4 at 500 MHz.	**S11**
**11**	Figure S10. HSQC spectrum of **1** and **2** in methanol-d_4 at 500 MHz.	**S12**
**12**	Figure S11. ROESY spectrum of **1** and **2** in methanol-d_4 at 500 MHz.	**S13**
**13**	Figure S12. LR-ESI-MS of **3**.	**S14**
**14**	Figure S13. HR-ESI-MS of **3**.	**S15**
**15**	Figure S14. ^{1}H NMR spectrum of **3** in methanol-d_4 at 500 MHz.	**S16**
**16**	Figure S15. ^{13}C NMR spectrum of **3** in methanol-d_4 at 125 MHz.	**S17**
**17**	Figure S16. ^{1}H-^{1}H COSY spectrum of **3** in methanol-d_4 at 500 MHz.	**S18**
**18**	Figure S17. HMBC spectrum of **3** in methanol-d_4 at 500 MHz.	**S19**
**19**	Figure S18. HSQC spectrum of **3** in methanol-d_4 at 500 MHz.	**S20**
**20**	Figure S19. ROESY spectrum of **3** in methanol-d_4 at 500 MHz.	**S21**
**21**	Figure S20. LR-ESI-MS of **4**.	**S22**
**22**	Figure S21. HR-ESI-MS of **4**.	**S23**
**23**	Figure S22. ^{1}H NMR spectrum of **4** in methanol-d_4 at 500 MHz.	**S24**
**24**	Figure S23. ^{13}C NMR spectrum of **4** in methanol-d_4 at 125 MHz.	**S25**
**25**	Figure S24. ^{1}H-^{1}H COSY spectrum of **4** in methanol-d_4 at 500 MHz.	**S26**
**26**	Figure S25. HMBC spectrum of **4** in methanol-d_4 at 500 MHz.	**S27**
**27**	Figure S26. HSQC spectrum of **4** in methanol-d_4 at 500 MHz.	**S28**
**28**	Figure S27. ROESY spectrum of **4** in methanol-d_4 at 500 MHz.	**S29**
**29**	Figure S28. LR-ESI-MS of **5**.	**S30**
**30**	Figure S29. HR-ESI-MS of **5**.	**S31**
**31**	Figure S30. ^{1}H NMR spectrum of **5** in methanol-d_4 at 500 MHz.	**S32**
**32**	Figure S31. ^{13}C NMR spectrum of **5** in methanol-d_4 at 125 MHz.	**S33**
**33**	Figure S32. ^{1}H-^{1}H COSY spectrum of **5** in methanol-d_4 at 500 MHz.	**S34**
**34**	Figure S33. HMBC spectrum of **5** in methanol-d_4 at 500 MHz.	**S35**
**35**	Figure S34. HSQC spectrum of **5** in methanol-d_4 at 500 MHz.	**S36**
**36**	Figure S35. ROESY spectrum of **5** in methanol-d_4 at 500 MHz.	**S37**
**37**	Figure S36 LR-ESI-MS of **6**/**7**.	**S38**
**38**	Figure S37. HR-ESI-MS of **6**/**7**.	**S39**
**39**	Figure S38. ^{1}H NMR spectrum of **6** and **7** (1:3) in methanol-d_4 at 500 MHz.	**S40**
**40**	Figure S39. ^{13}C NMR spectrum of **6** and **7** (1:3) in methanol-d_4 at 125 MHz.	**S41**
**41**	Figure S40. ^{1}H-^{1}H COSY spectrum of **6** and **7** (1:3) in methanol-d_4 at 500 MHz.	**S42**
**42**	Figure S41. HMBC spectrum of **6** and **7** (1:3) in methanol-d_4 at 500 MHz.	**S43**
**43**	Figure S42. HSQC spectrum of **6** and **7** (1:3) in methanol-d_4 at 500 MHz.	**S44**
**44**	Figure S43. ROESY spectrum of **6** and **7** (1:3) in methanol-d_4 at 500 MHz.	**S45**

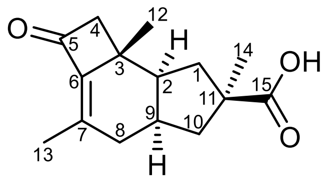

Tricoprotoilldene A (**2**)

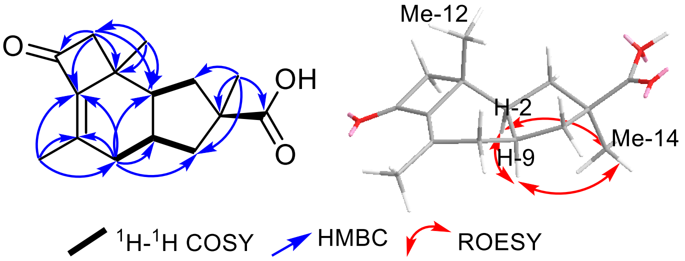

Figure S1. Key ^{1}H–^{1}H COSY, HMBC and ROESY correlations of **2**.

Table S1. ^{1}H and ^{13}C NMR data of compound (**2**) and tricoprotoilludene A.[a]

	**2**		Tricoprotoilludene A[a]	
pos	δ_C,[b] type	δ_H[b] (multi, *J* [Hz])	δ_C,[c] type	δ_H[c] (multi, *J* [Hz])
1	38.5, CH_2	*α* 1.75 overlapped<br>*β* 2.17 overlapped	37.8, CH_2	*α* 1.51 dd (13.0, 9.7)<br>*β* 2.37 overlapped
2	47.9, CH	2.35 overlapped	47.2, CH	2.29 br t (9.8)
3	38.0, C		36.5, C	
4	61.7, CH_2	*α* 2.65 dt (16.5, 0.9)<br>*β* 2.74 d (16.5)	60.9, CH_2	*α* 2.68 d (16.5)<br>*β* 2.74 d (16.5)
5	199.2, CO		197.0, CO	
6	151.8, C		150.6, C	
7	145.8, C		143.1, C	
8	36.5, CH_2	*α* 1.94 dd (14.9, 10.3)<br>*β* 2.34 dd (14.9, 6.8)	35.4, CH_2	*α* 1.82 dd (14.7, 9.9)<br>*β* 2.25 dd (14.7, 6.6)
9	42.7, CH	2.55 m	42.0, CH	2.45 overlapped
10	45.5, CH_2	*α* 1.74 overlapped<br>*β* 1.86 overlapped	45.1, CH_2	*α* 1.17 overlapped<br>*β* 2.41 overlapped
11	51.1, C		51.0, C	
12	20.9, CH_3	1.19 s	20.4, CH_3	1.16 s
13	20.5, CH_3	2.02 d (0.4)	20.4, CH_3	2.01 s
14	23.8, CH_3	1.28 s	24.8, CH_3	1.41 s
15	181.3, CO		182.0, CO	

[a] Kanehara, R.; Tonouchi, A.; Konno, K.; Hashimoto, M. Cyclohumulanoid Sesquiterpenes from the Culture Broth of the Basidiomycetous Fungus *Daedaleopsis tricolor*. *Molecules* **2021**, 26, 4364; https://doi.org/10.3390/molecules26144364

[b] Measured in methanol-d_4 at 125 MHz for ^{13}C and 500 MHz for ^{1}H.

[c] Measured in chloroform-*d* at 125 MHz for ^{13}C and 500 MHz for ^{1}H.

Display Report

Analysis Info

Analysis Name	S:\DATA\AmaZon\wsu20_Winnier Sum Chemutai\IHI CRUDE\IHI 760 Compounds\IHI 760 R1F5F1_GE7_01_51244.d	Acquisition Date	28.09.2023 07:22:22
Method	51244.m	Operator	tti
Sample Name	IHI 760 R1F5F1	Instrument	amaZon speed
Comment			

Acquisition Parameter

Ion Source Type	ESI	Ion Polarity	Negative	Alternating Ion Polarity	on
Mass Range Mode	UltraScan	Scan Begin	100 m/z	Scan End	2000 m/z
Accumulation Time	3575 µs	RF Level	100 %	Trap Drive	78.0
SPS Target Mass	1000 m/z	Averages	6 Spectra		

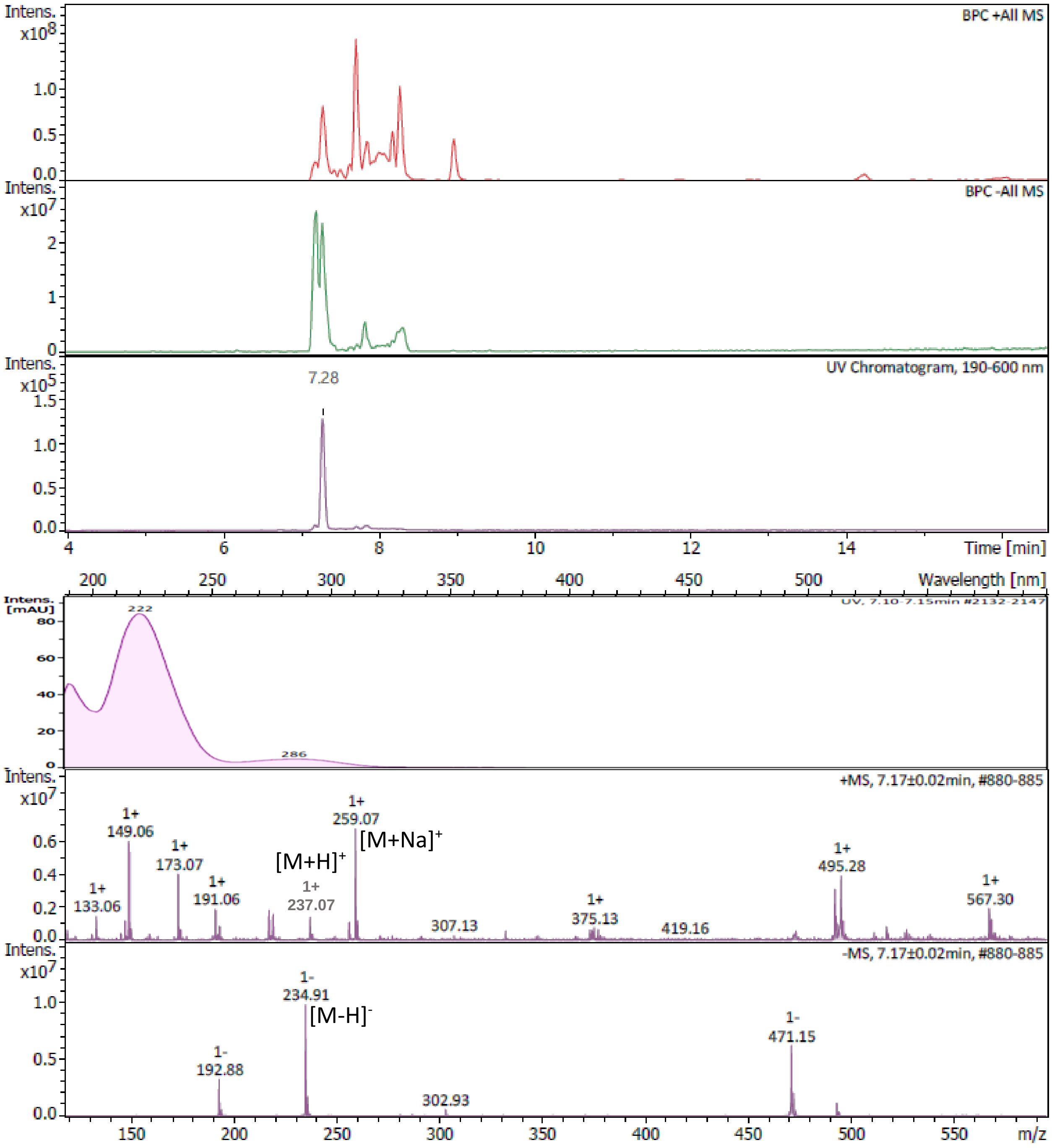

Bruker Compass DataAnalysis 6.1 printed: 19.03.2024 17:24:51 by: sel22 Page 1 of 1

Figure S2. LR-ESI-MS of **1**.

Generic Display Report

Analysis Info

Analysis Name	S:\PEOPLE\sel22_Sherif Elsayed\Phellinus\IHI 760\AmaZon\IHI 760 R1F5F1_GE7_01_51244.d	Acquisition Date	28.09.2023 07:22:22
Method	51244.m	Operator	tti
Sample Name	IHI 760 R1F5F1	Instrument	amaZon speed
Comment			

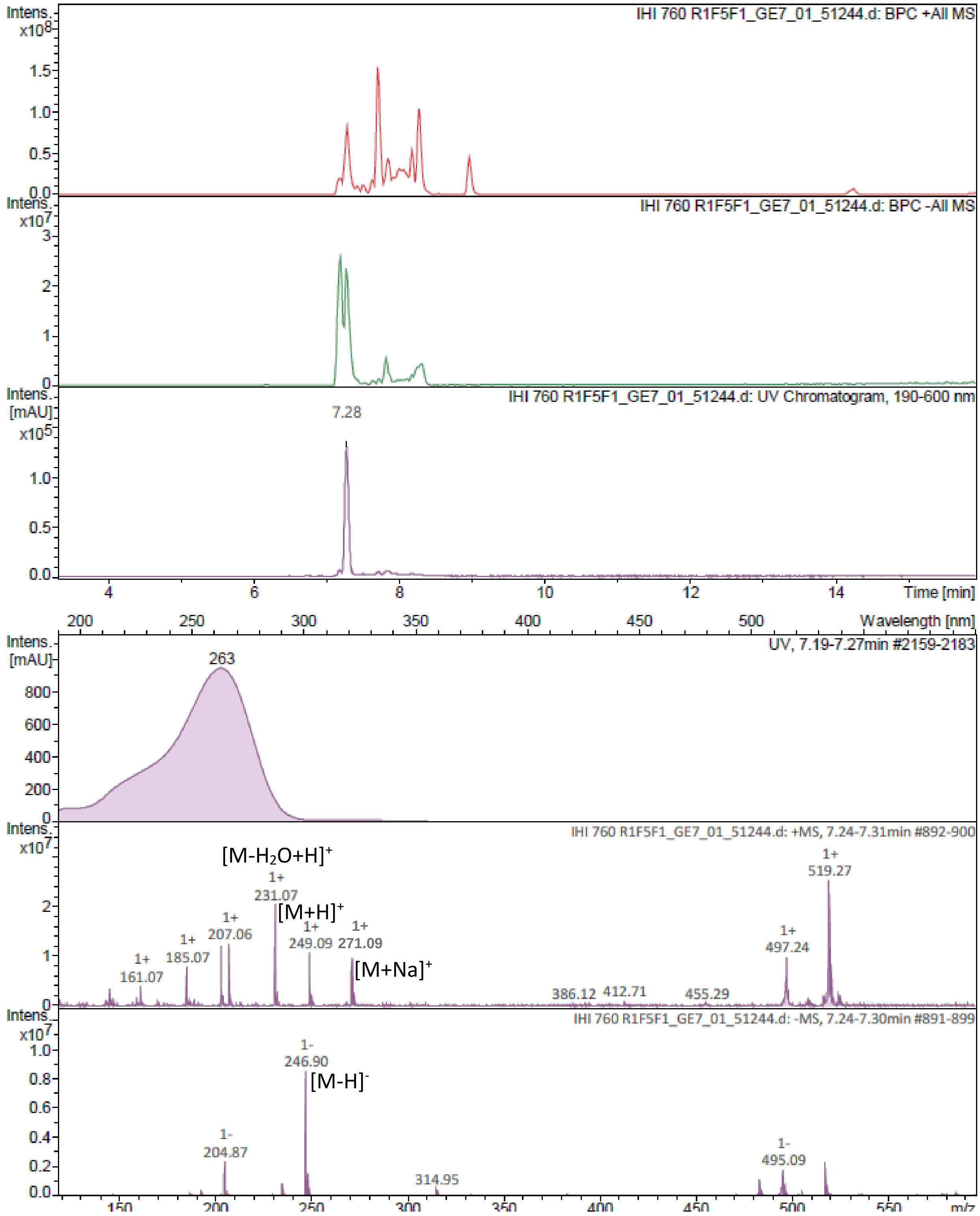

Bruker Compass DataAnalysis 4.4 printed: 19.10.2023 15:29:49 by: sel22 Page 1 of 1

Figure S3. LR-ESI-MS of **2**.

Display Report

Analysis Info

		Acquisition Date	29.09.2023 13:25:33
Analysis Name	S:\PEOPLE\sel22_Sherif Elsayed\Phellinus\IHI 760\MaXis\IHI 760 R2F6_13_01_13358.d		
Method	pos_säure_10000_screening_ms_100_2500_line.m	Operator	ate06
Sample Name	IH 760 R2F3	Instrument	maXis
Comment	Screening01<br>Waters Acquity UPLC BEH C_18 1,7um 2.1x50mm		

Acquisition Parameter

Ion Polarity Positive

SPS Target Mass

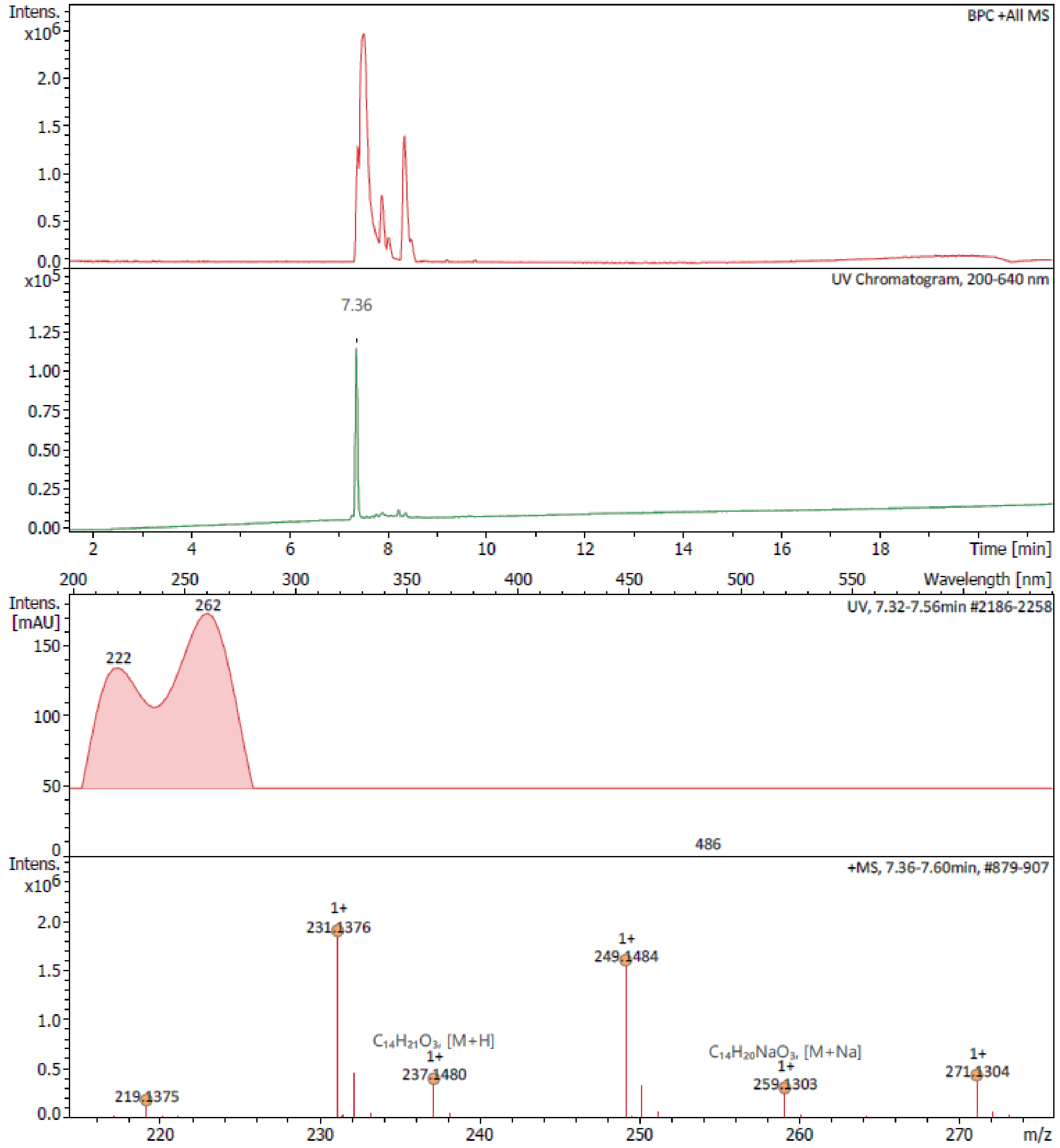

Bruker Compass DataAnalysis 6.1 printed: 07.12.2023 09:47:06 by: sel22 Page 1 of 1

Figure S4. HR-ESI-MS of **1**.

Generic Display Report

Analysis Info

Analysis Name	S:\PEOPLE\sel22_Sherif Elsayed\Phellinus\IHI 760\MaXis\IHI 760 R2F6_13_01_13358.d	Acquisition Date	29.09.2023 13:25:33
Method	pos_säure_10000_screening_ms_100_2500_line.m	Operator	ate06
Sample Name	IH 760 R2F3	Instrument	maXis
Comment	Screening01<br>Waters Acquity UPLC BEH C_18 1,7um 2.1x50mm		

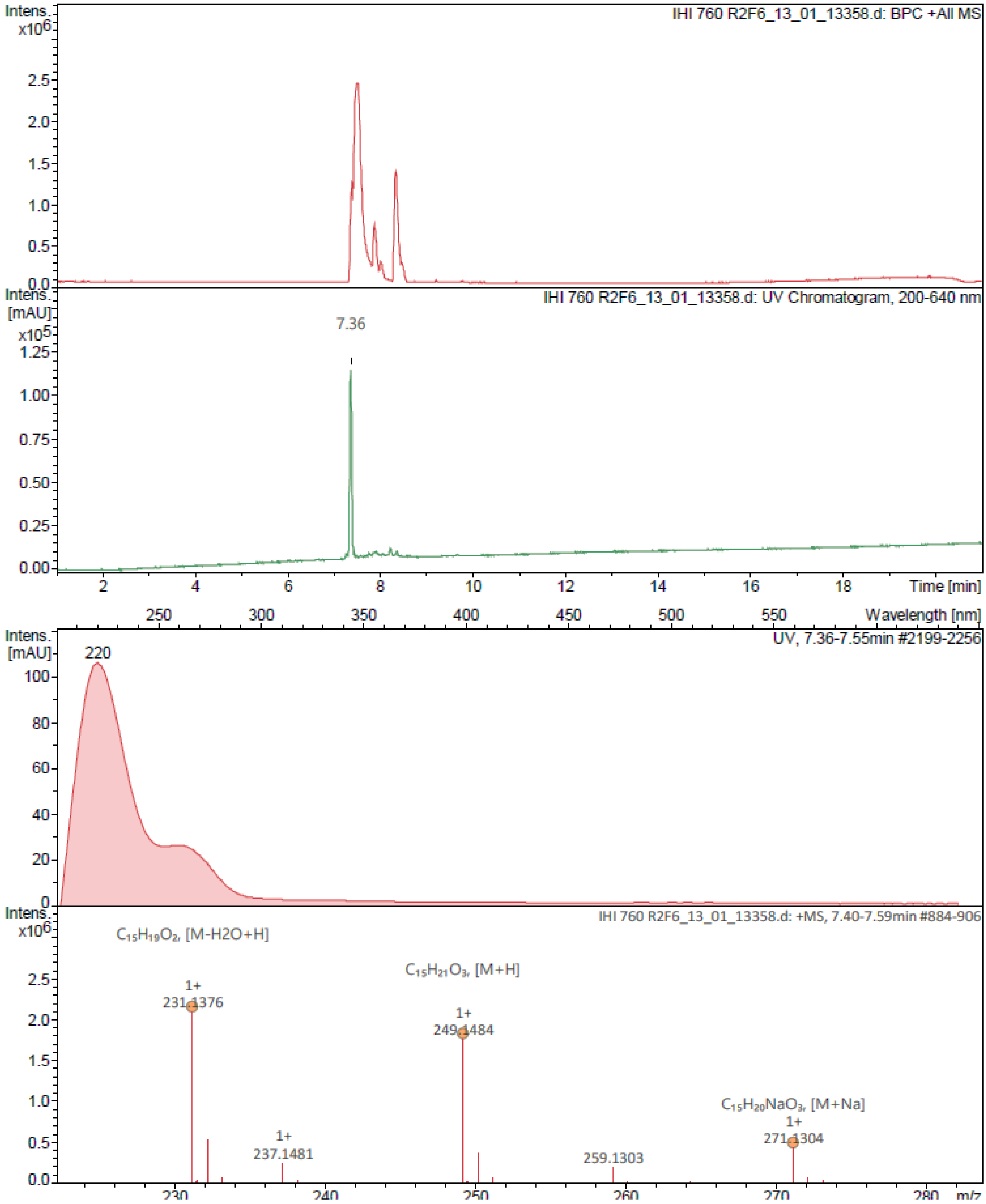

Figure S5. HR-ESI-MS of **2**.

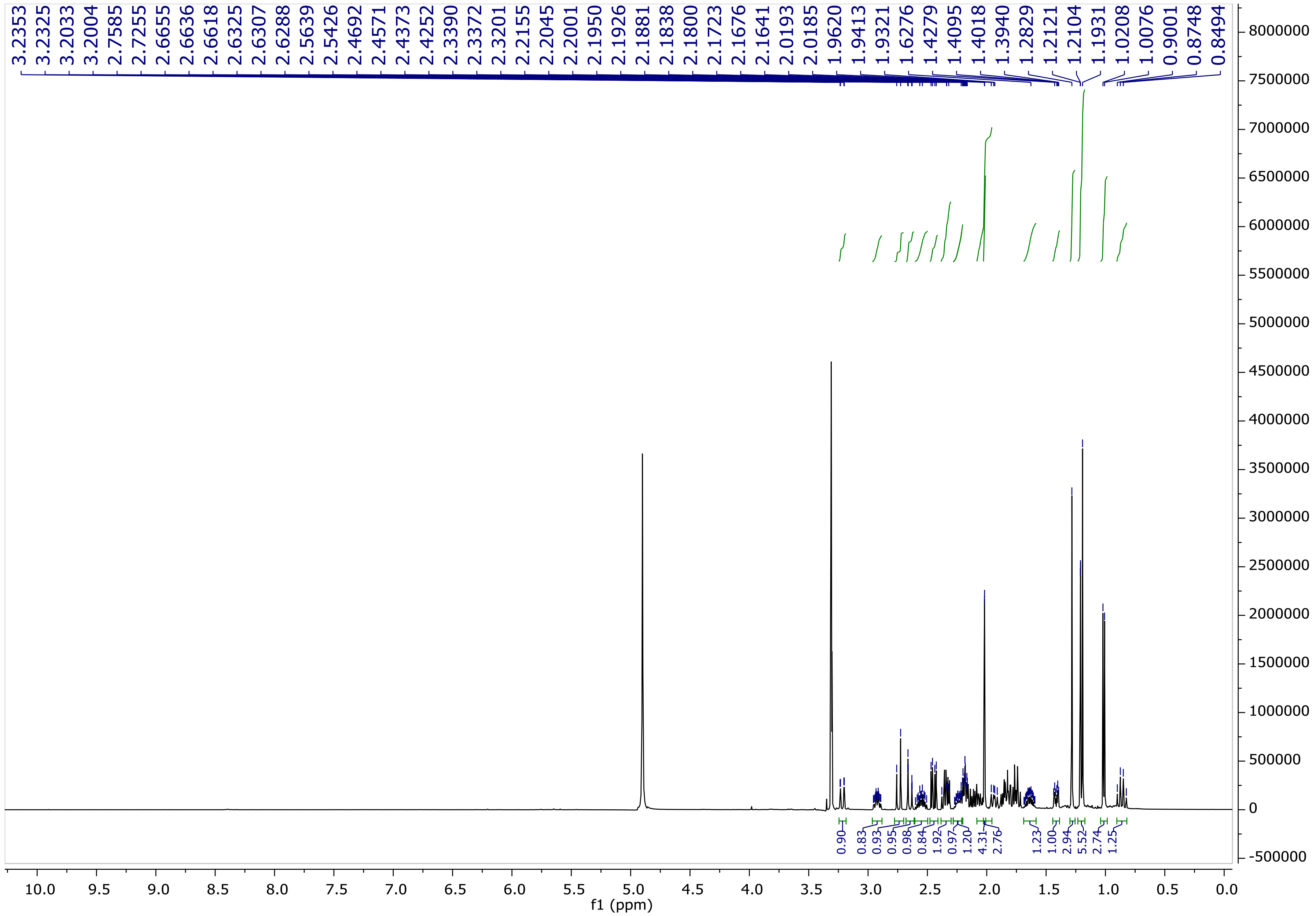

Figure S6. ^{1}H NMR spectrum of **1** and **2** in methanol-d_4 at 500 MHz.

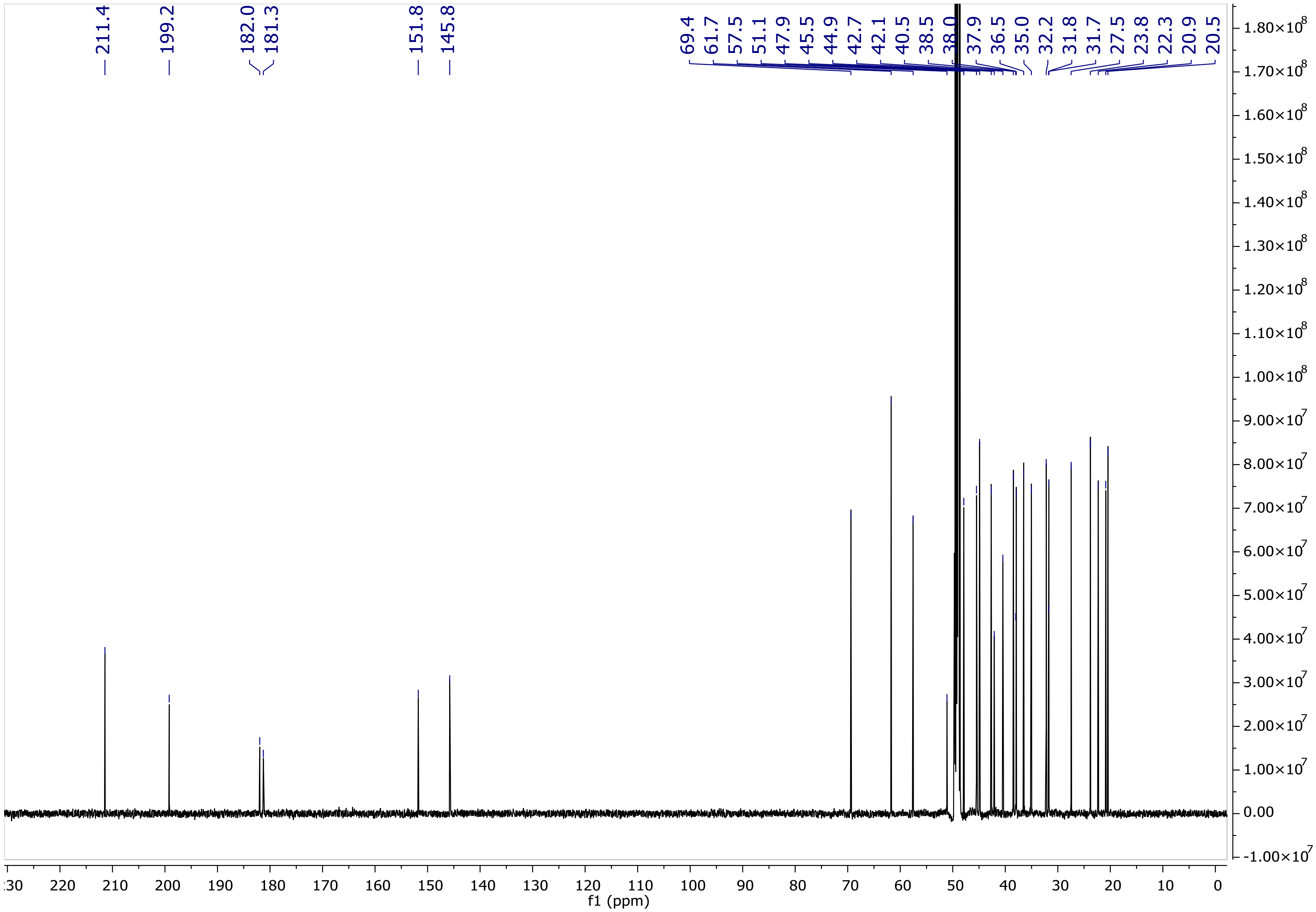

Figure S7. ^{13}C NMR spectrum of **1** and **2** in methanol-d_4 at 125 MHz.

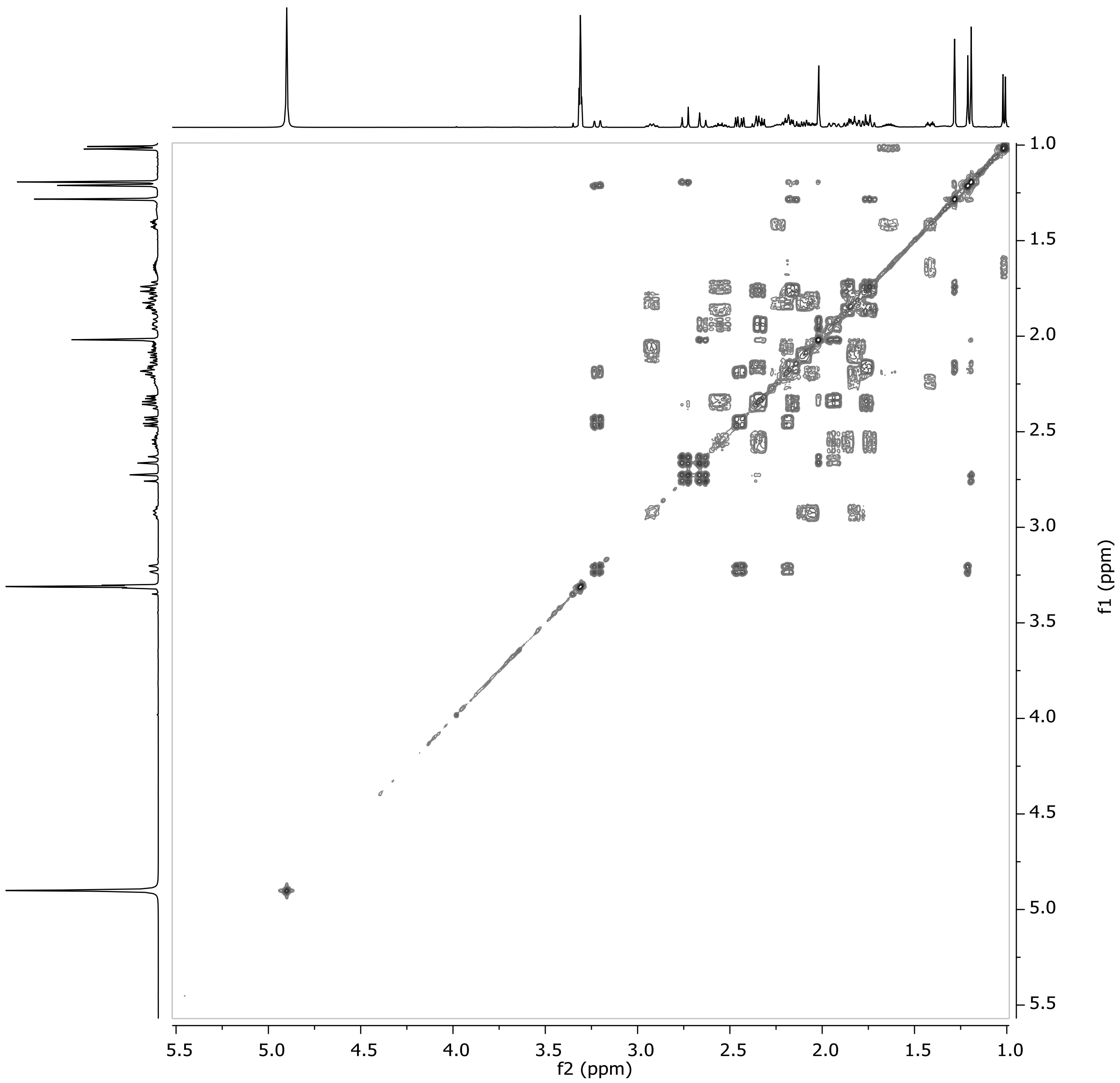

Figure S8. ^{1}H-^{1}H COSY spectrum of **1** and **2** in methanol-d_4 at 500 MHz.

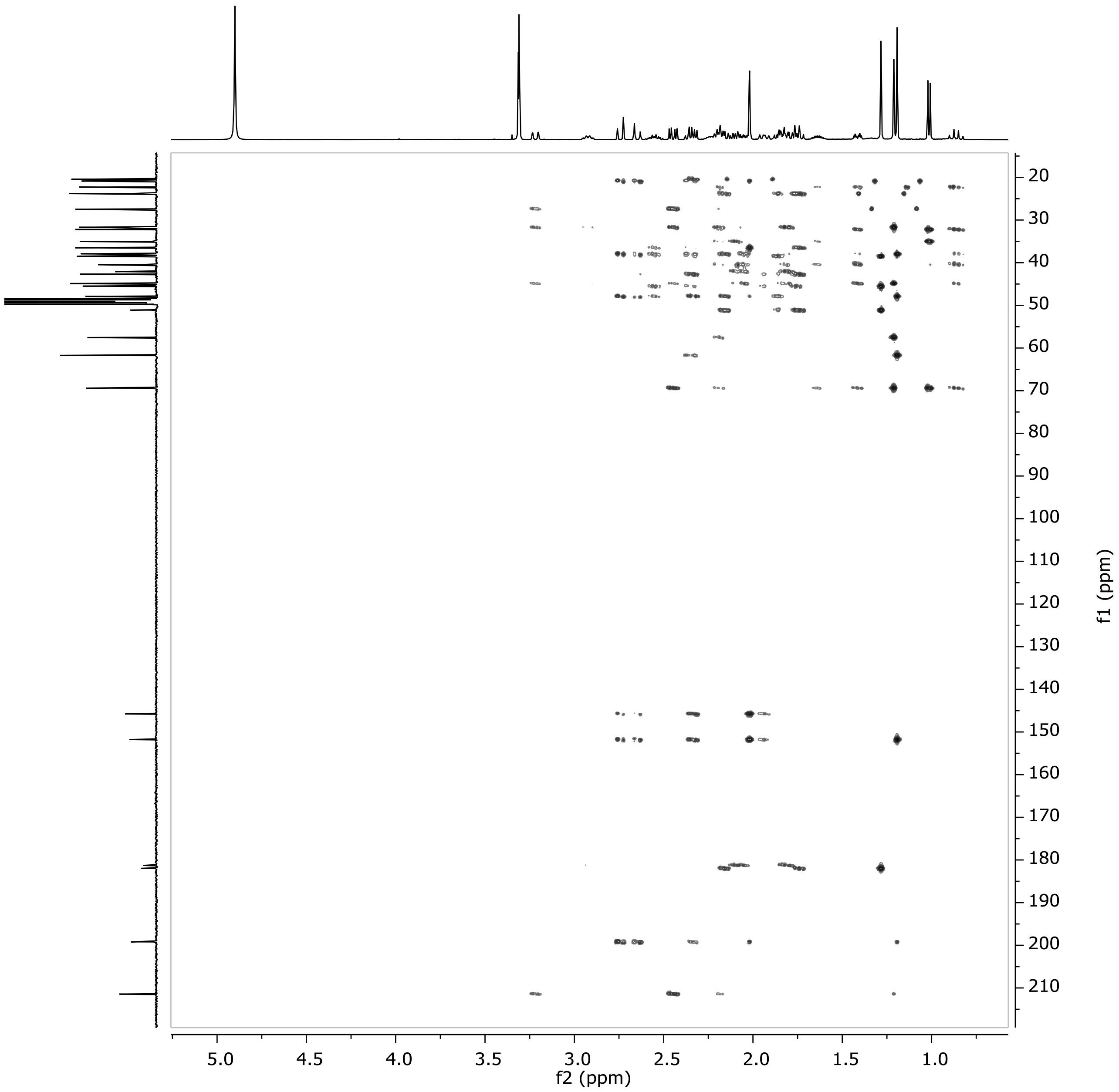

Figure S9. HMBC spectrum of **1** and **2** in methanol-d_4 at 500 MHz.

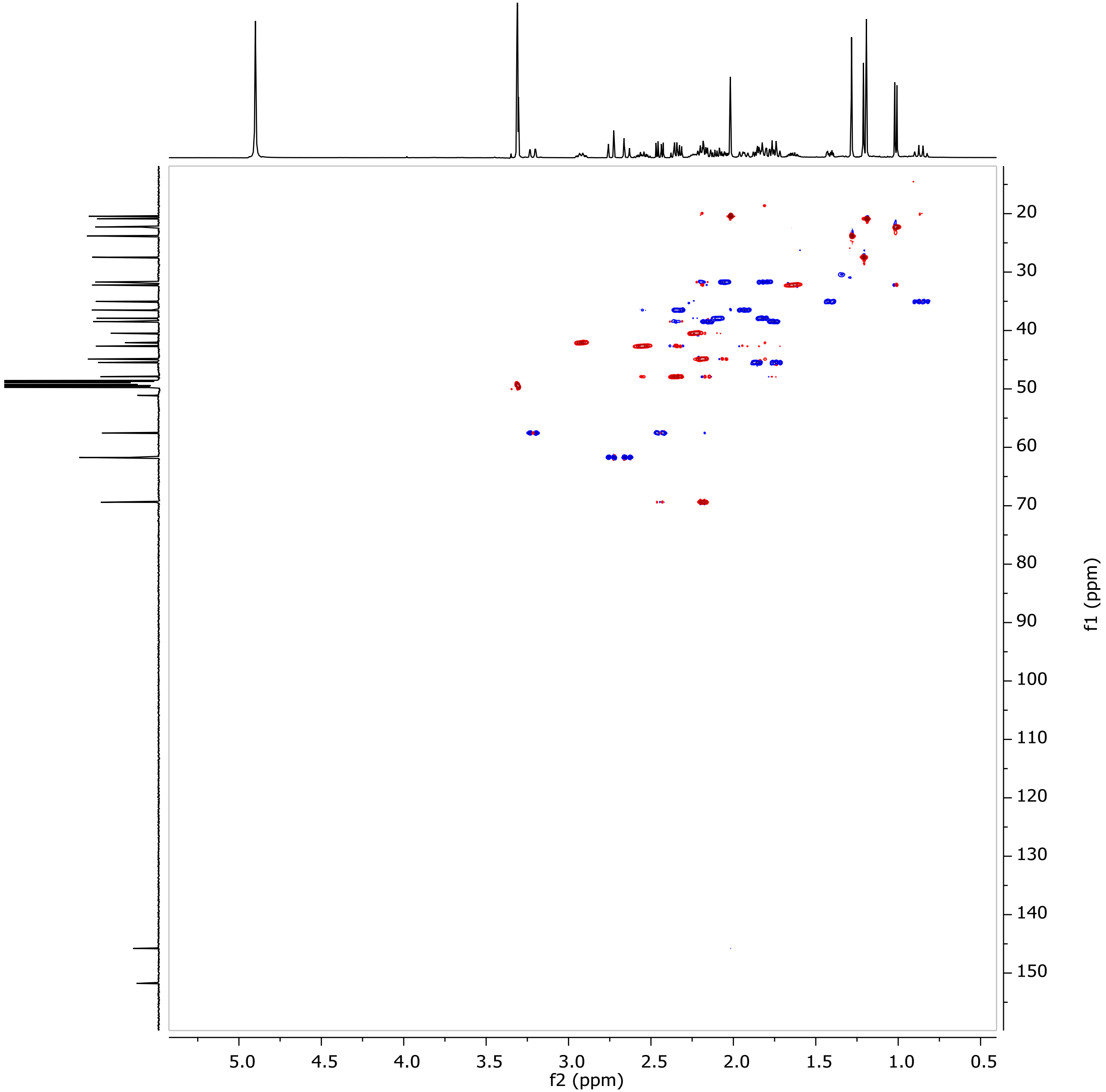

Figure S10. HSQC spectrum of **1** and **2** in methanol-d_4 at 500 MHz.

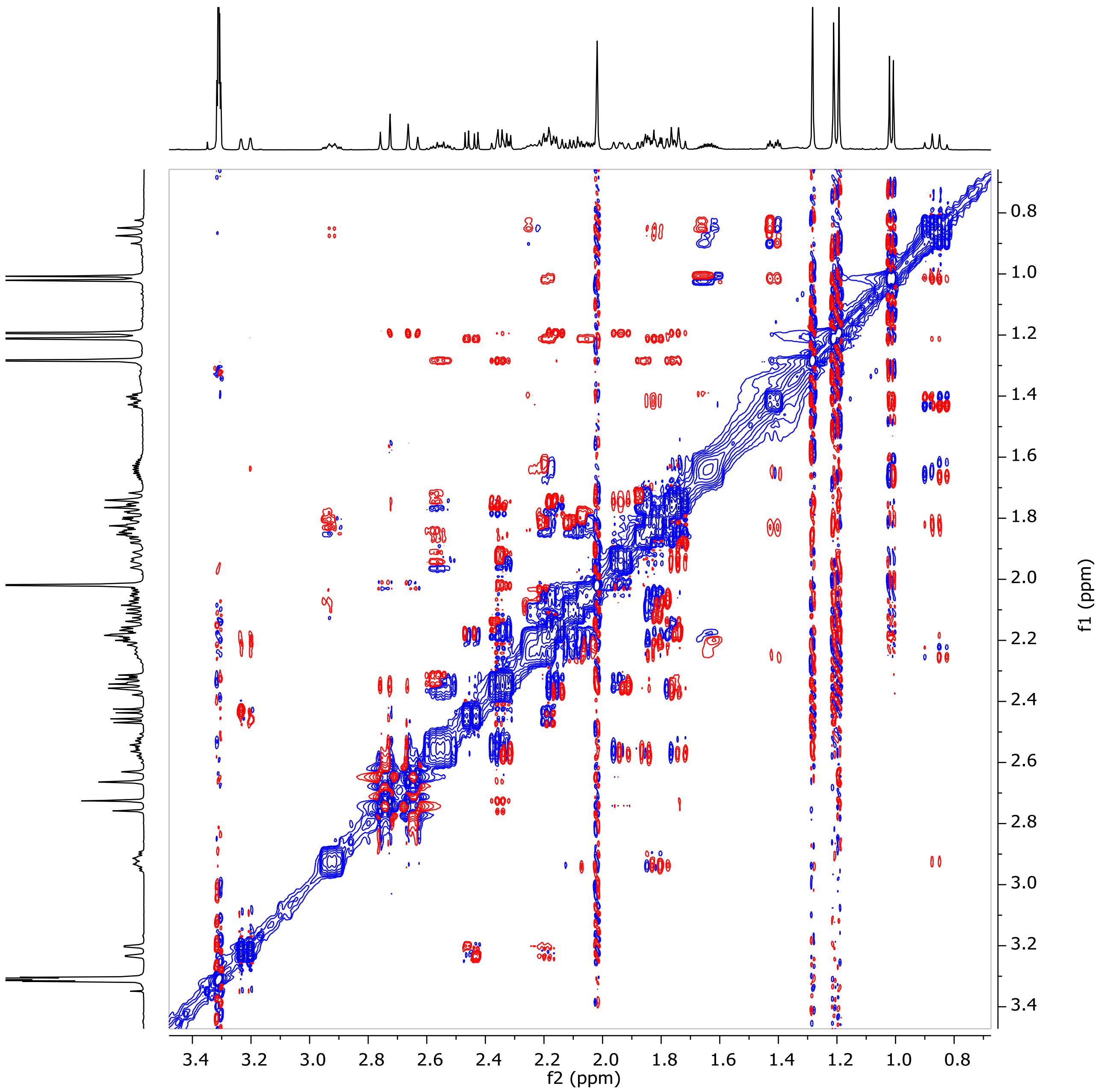

Figure S11. ROESY spectrum of **1** and **2** in methanol-d_4 at 500 MHz.

Generic Display Report

Analysis Info

Analysis Name	S:\PEOPLE\sel22_Sherif Elsayed\Phellinus\IHI 760\AmaZon\IHI 760 R2F9_RD1_01_51153.d	Acquisition Date	26.09.2023 00:19:58
Method	51153.m	Operator	tti
Sample Name	IHI 760 R2F9	Instrument	amaZon speed
Comment			

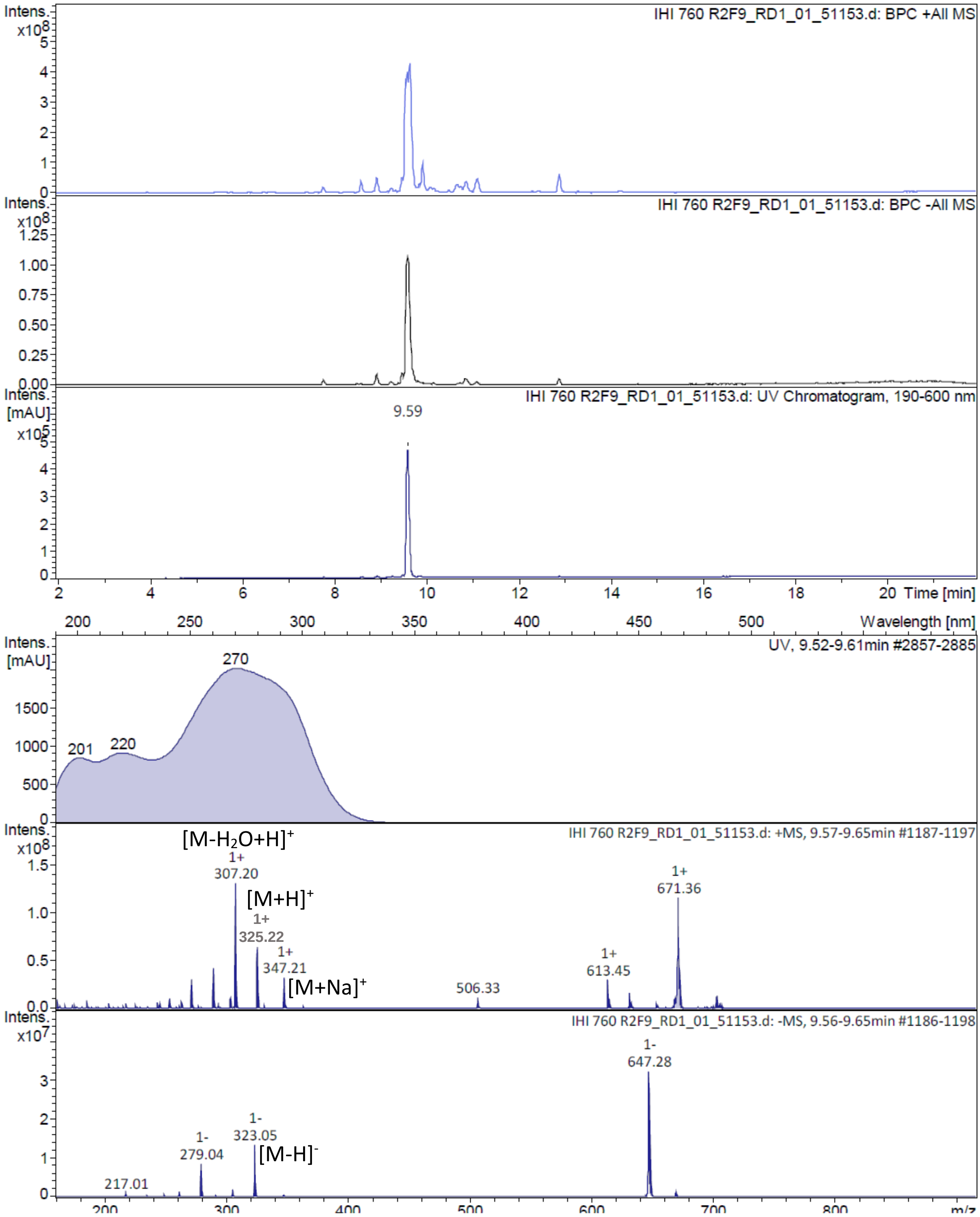

Bruker Compass DataAnalysis 4.4 printed: 19.10.2023 15:35:27 by: sel22 Page 1 of 1

Figure S12. LR-ESI-MS of **3**.

Generic Display Report

**Analysis Info**		Acquisition Date	29.09.2023 14:27:28
Analysis Name	S:\PEOPLE\sel22_Sherif Elsayed\Phellinus\IHI 760\MaXis\IHI 760 R2F9_15_01_13360.d		
Method	pos_säure_10000_screening_ms_100_2500_line.m	Operator	ate06
Sample Name	IH 760 R2F9	Instrument	maXis
Comment	Screening01<br>Waters Acquity UPLC BEH C_18 1,7um 2.1x50mm		

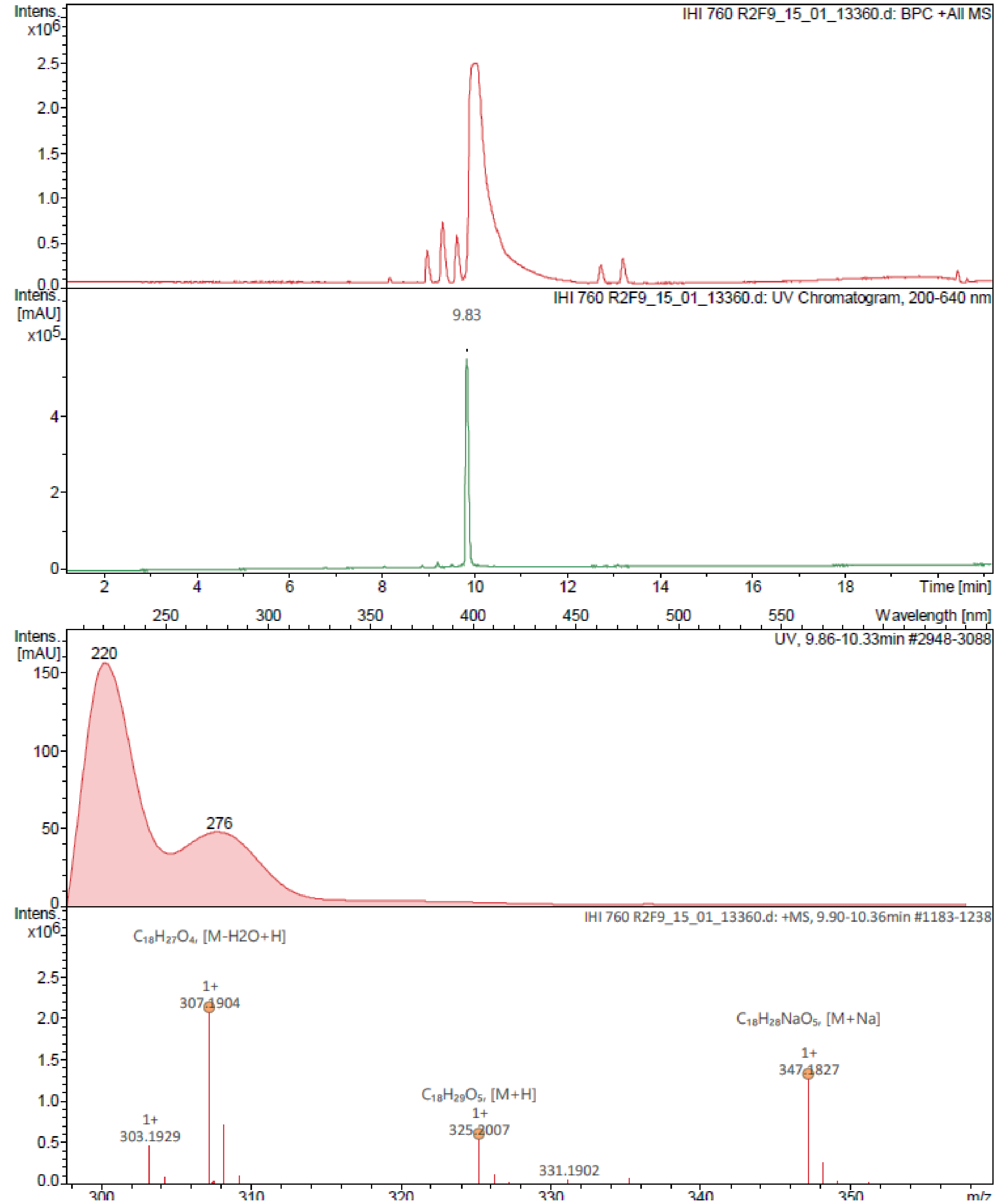

Bruker Compass DataAnalysis 4.4 printed: 19.10.2023 18:27:15 by: sel22 Page 1 of 1

Figure S13. HR-ESI-MS of **3**.

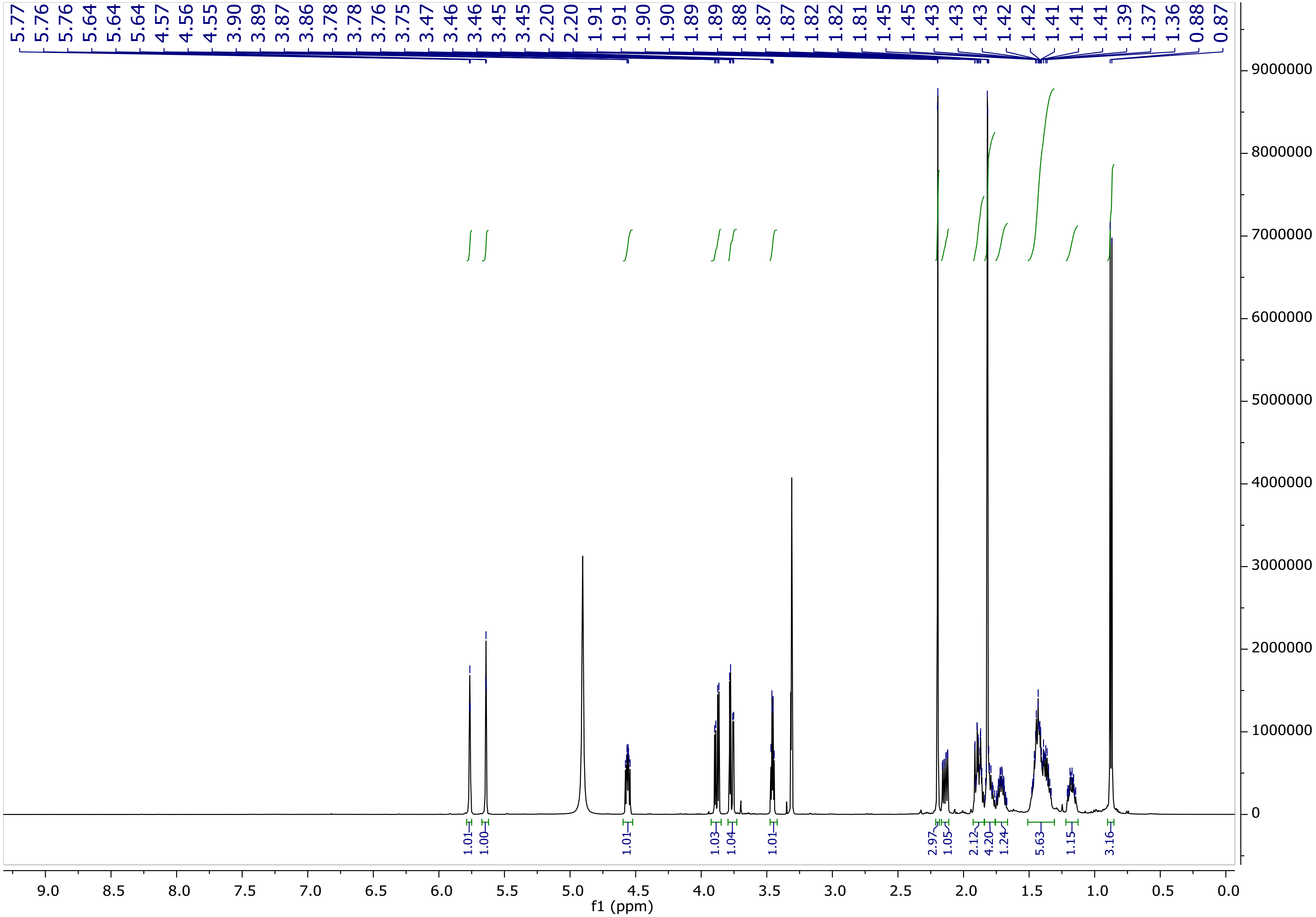

Figure S14. ^{1}H NMR spectrum of **3** in methanol-d_4 at 500 MHz.

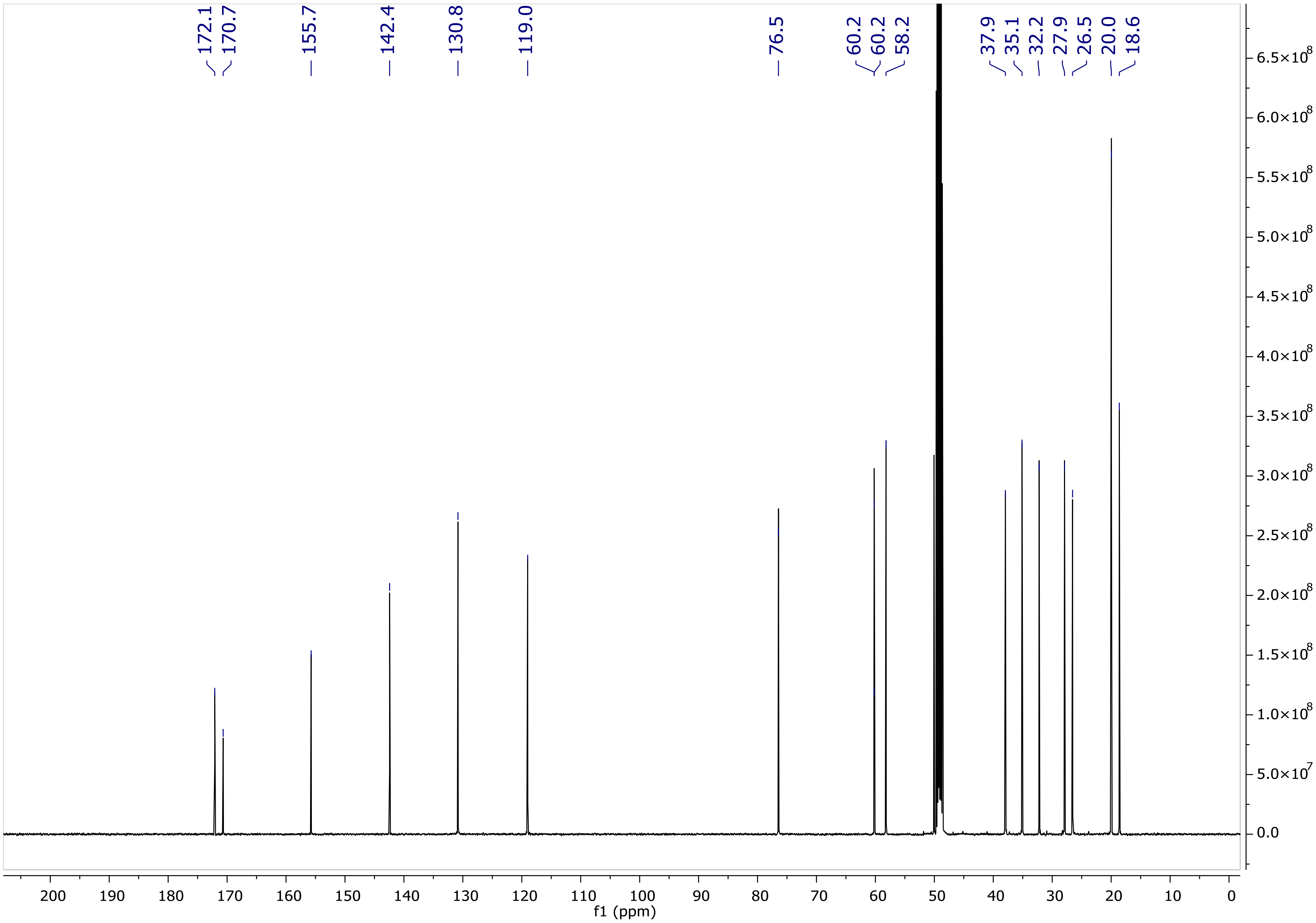

Figure S15. ^{13}C NMR spectrum of **3** in methanol-d_4 at 125 MHz.

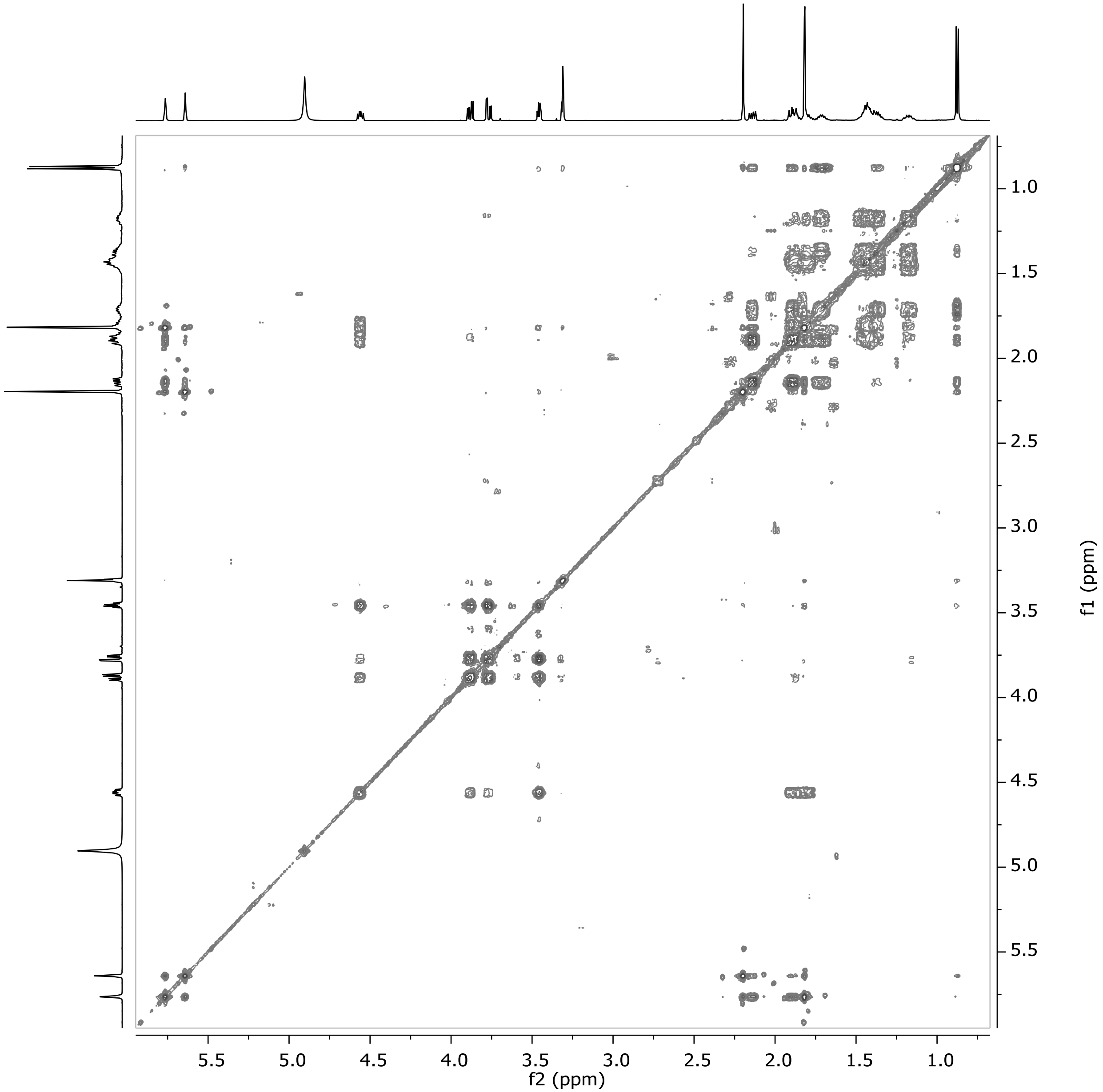

Figure S16. ^{1}H-^{1}H COSY spectrum of **3** in methanol-d_4 at 500 MHz.

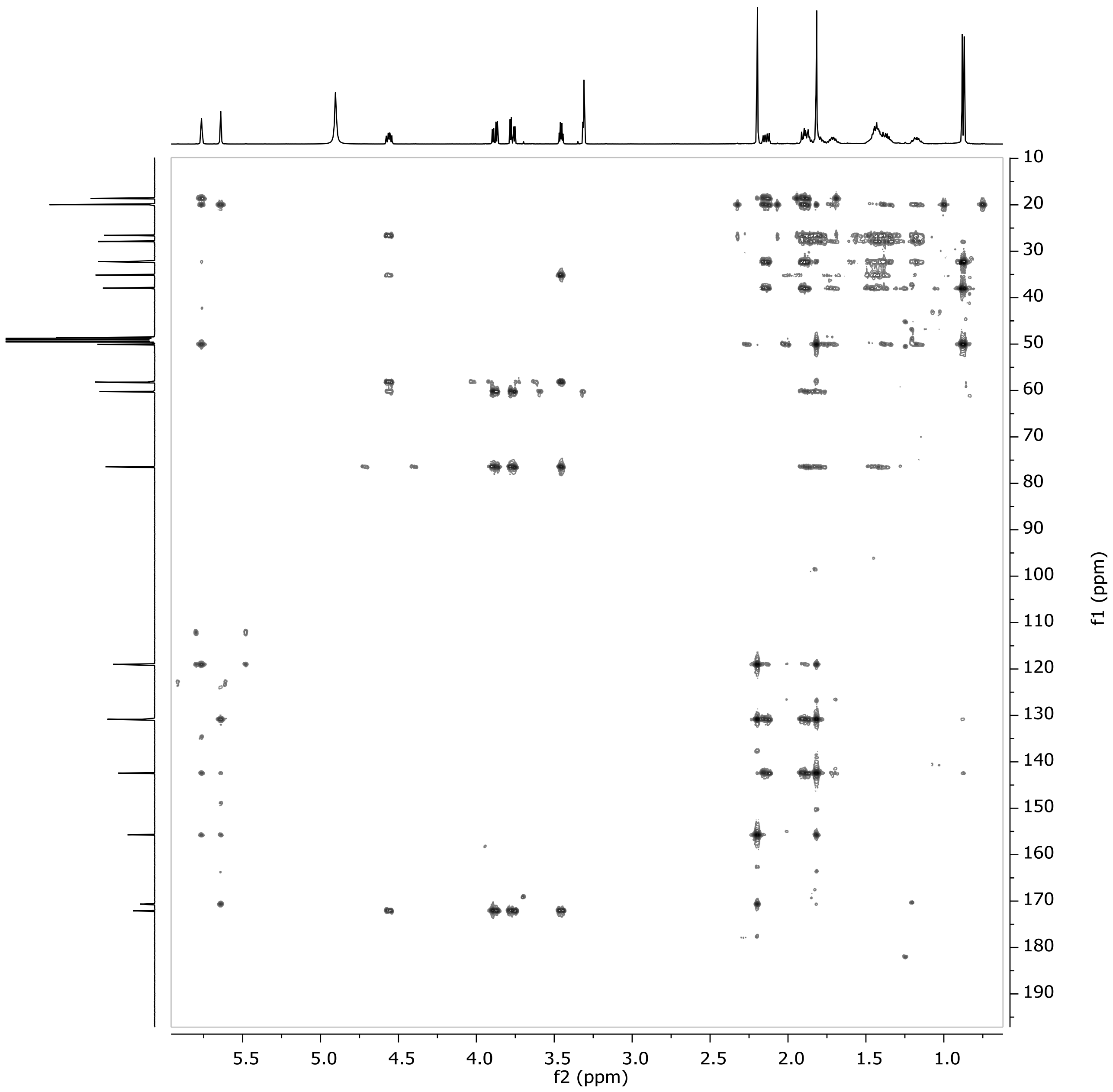

Figure S17. HMBC spectrum of **3** in methanol-d_4 at 500 MHz.

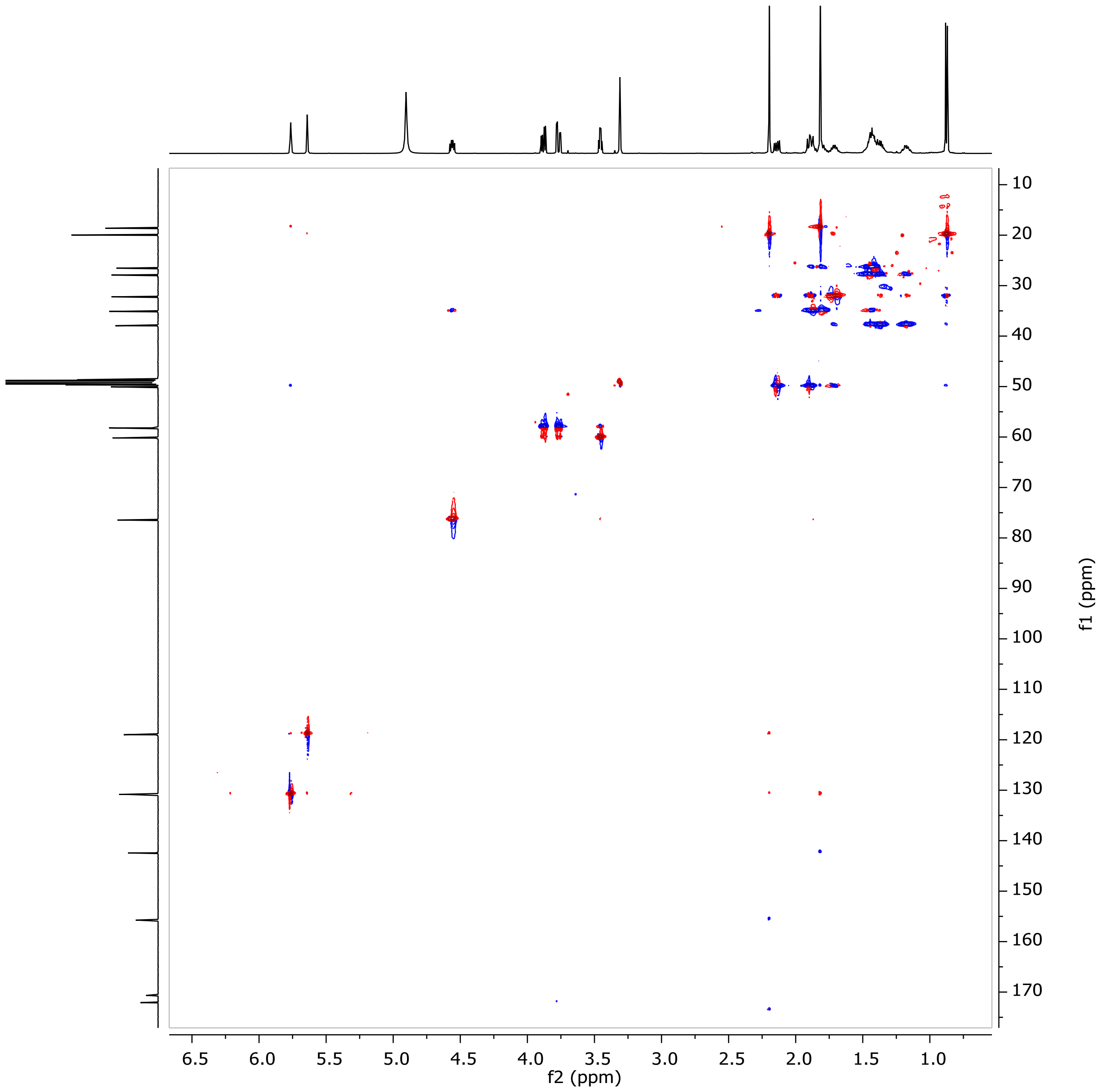

Figure S18. HSQC spectrum of **3** in methanol-d_4 at 500 MHz.

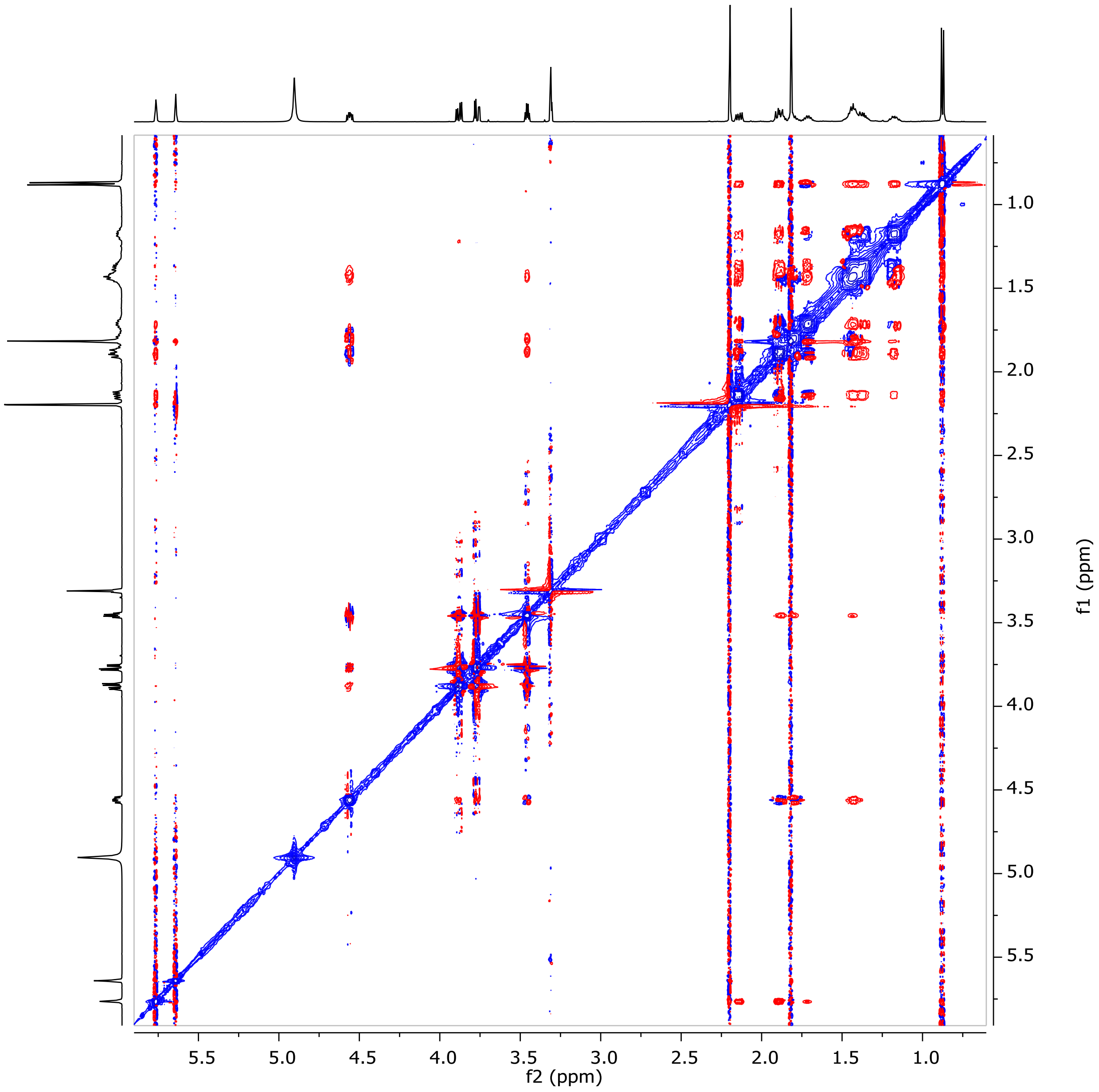

Figure S19. ROESY spectrum of **3** in methanol-d_4 at 500 MHz.

Generic Display Report

Analysis Info

		Acquisition Date	25.09.2023 21:55:01
Analysis Name	S:\PEOPLE\sel22_Sherif Elsayed\Phellinus\IHI 760\AmaZon\IHI 760 R2F5_RC5_01_51149.d		
Method	51149.m	Operator	tti
Sample Name	IHI 760 R2F5	Instrument	amaZon speed
Comment			

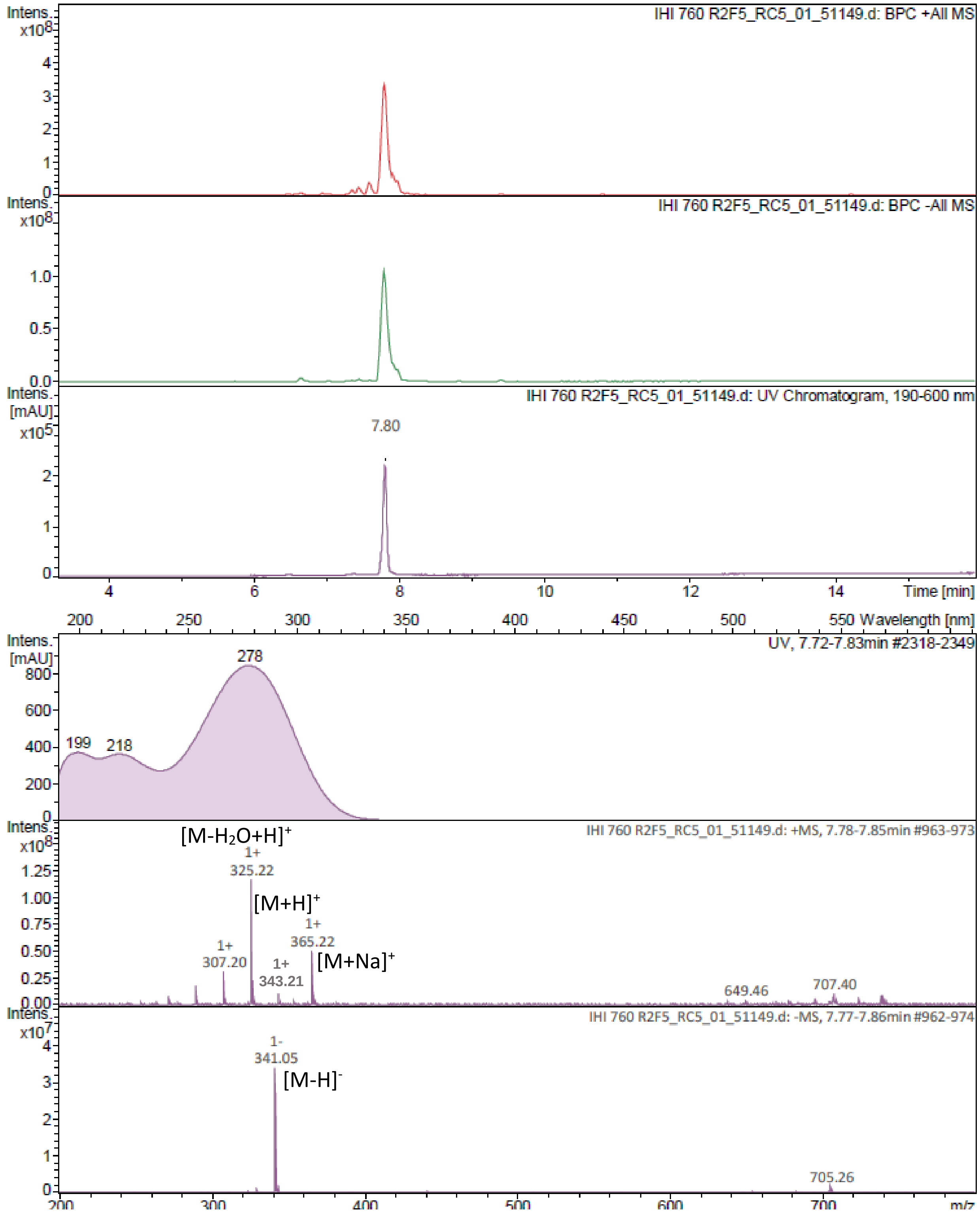

Bruker Compass DataAnalysis 4.4 printed: 19.10.2023 15:31:23 by: sel22 Page 1 of 1

Figure S20. LR-ESI-MS of **4**.

Generic Display Report

Analysis Info

Analysis Name	S:\PEOPLE\sel22_Sherif Elsayed\Phellinus\IHI 760\MaXis\IHI 760 R2F5_12_01_13357.d	Acquisition Date	29.09.2023 12:54:35
Method	pos_säure_10000_screening_ms_100_2500_line.m	Operator	ate06
Sample Name	IH 760 R2F6	Instrument	maXis
Comment	Screening01<br>Waters Acquity UPLC BEH C_18 1,7um 2.1x50mm		

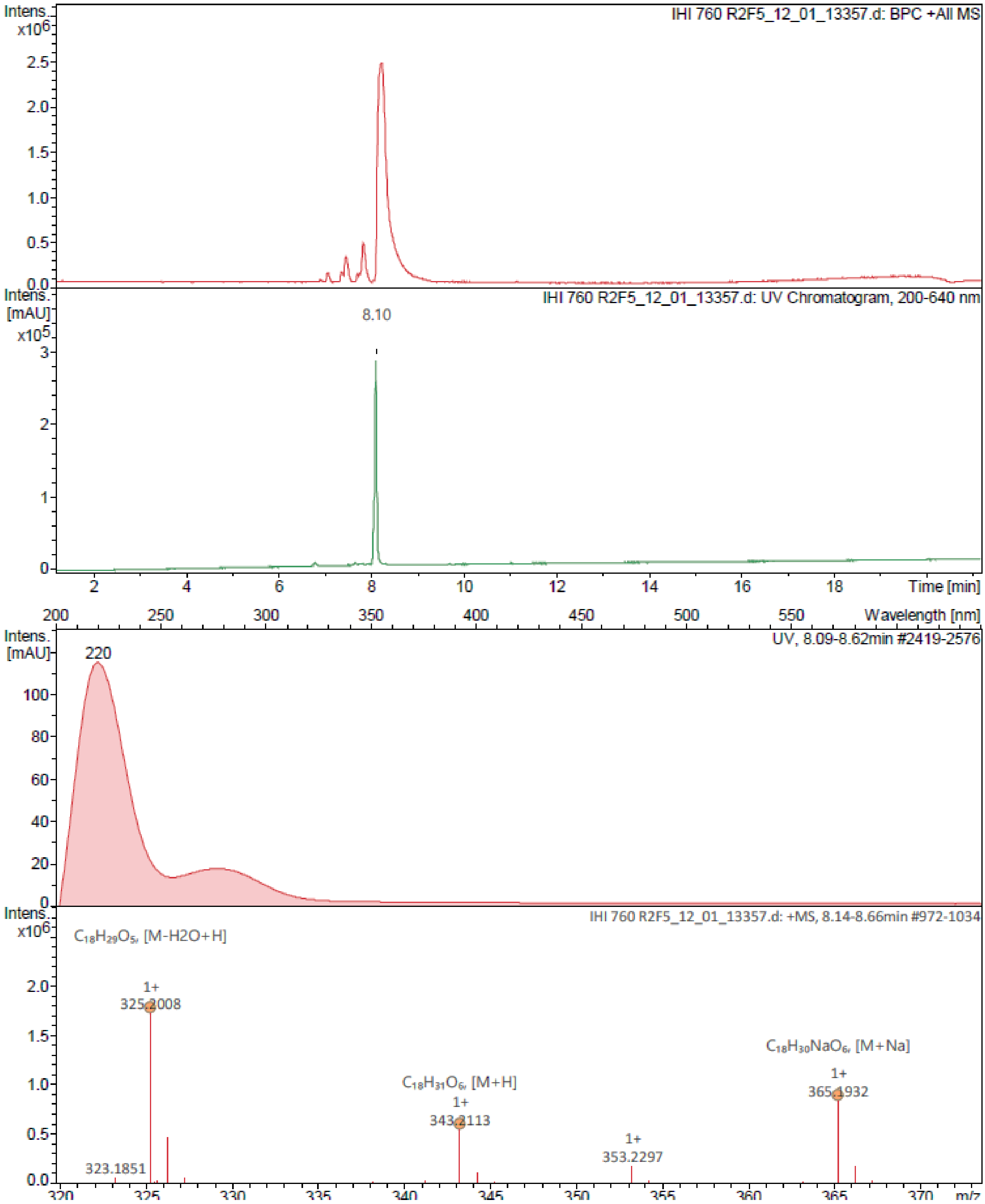

Figure S21. HR-ESI-MS of **4**.

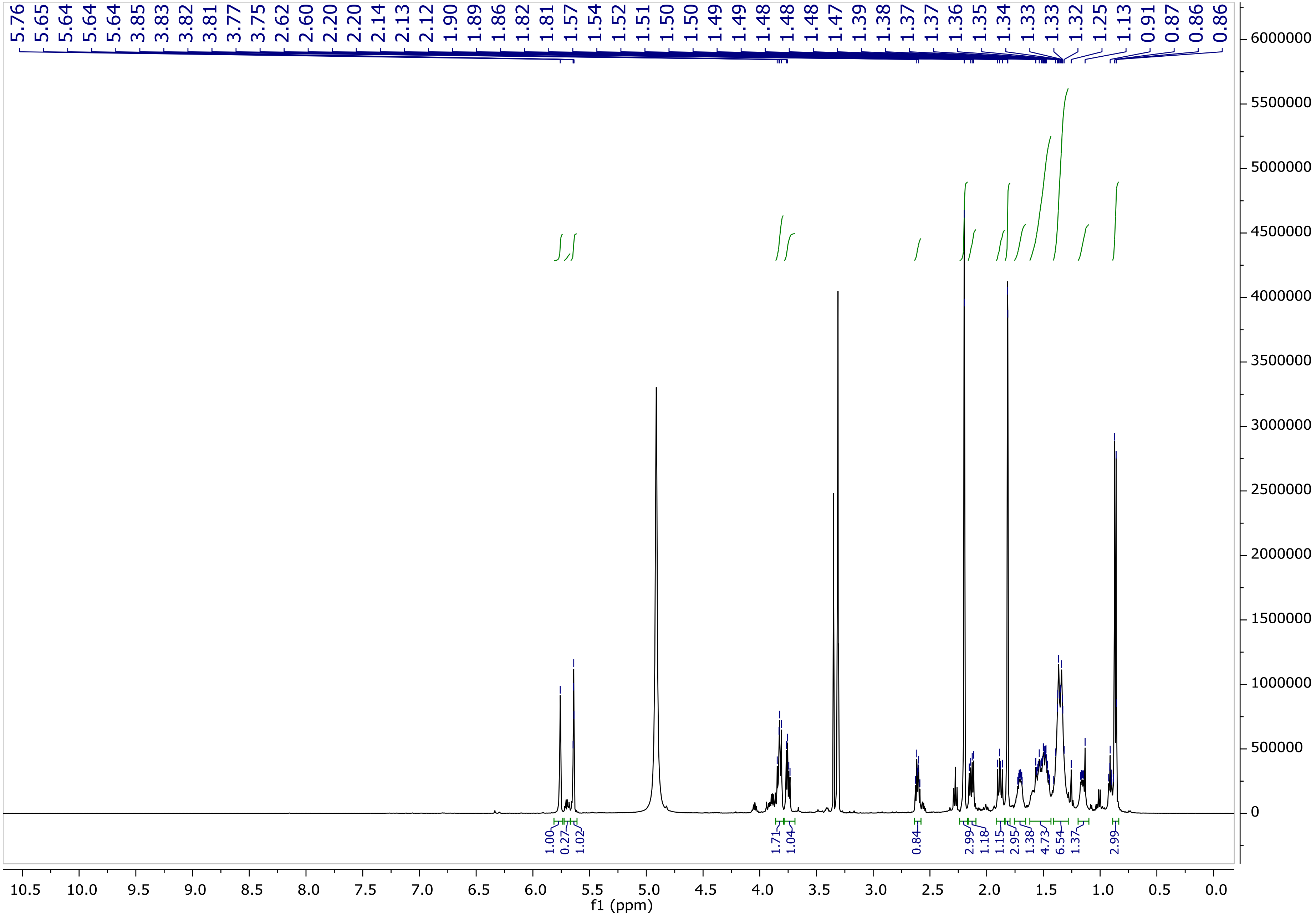

Figure S22. ^{1}H NMR spectrum of **4** in methanol-d_4 at 500 MHz.

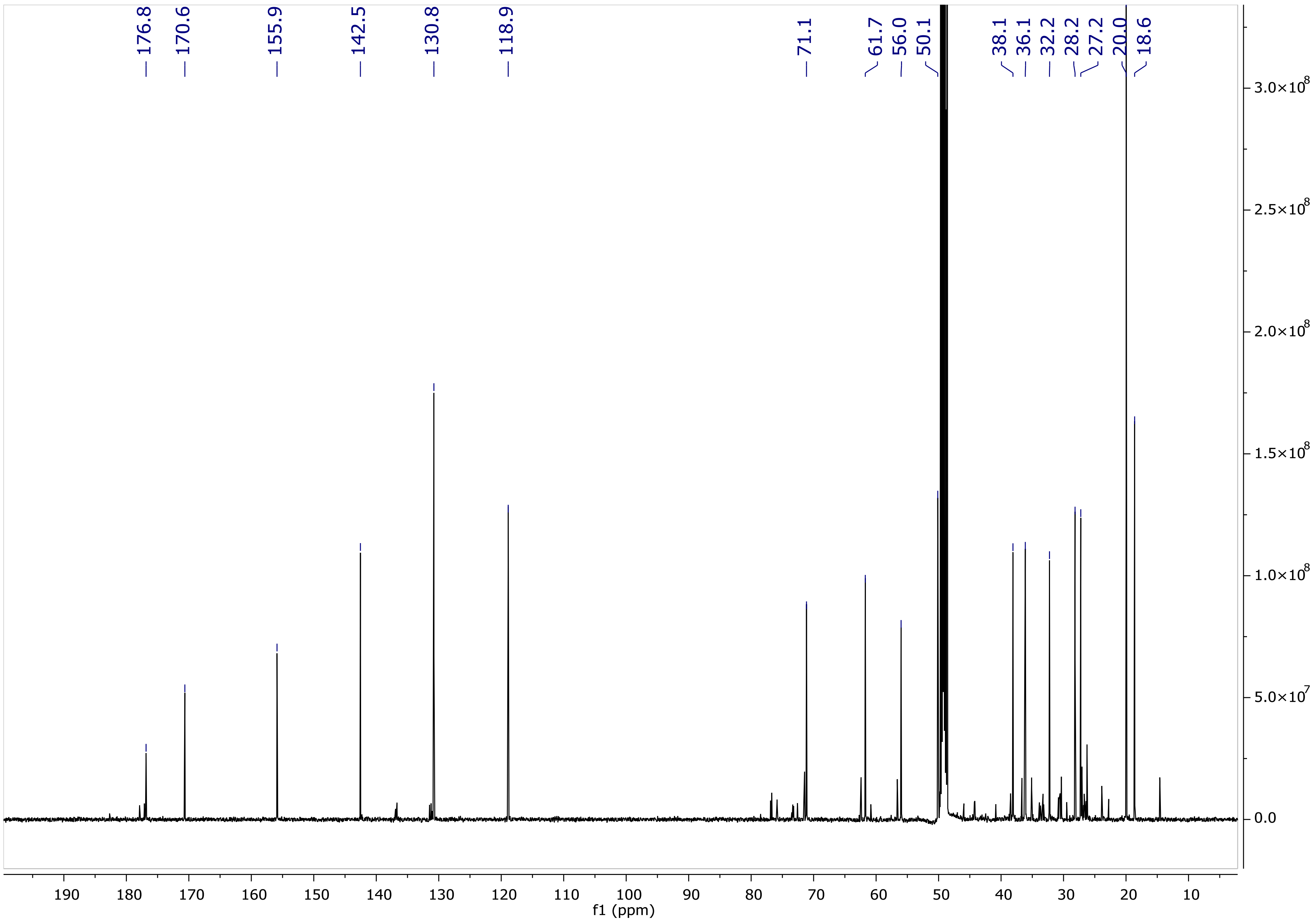

Figure S23. ^{13}C NMR spectrum of **4** in methanol-d_4 at 125 MHz.

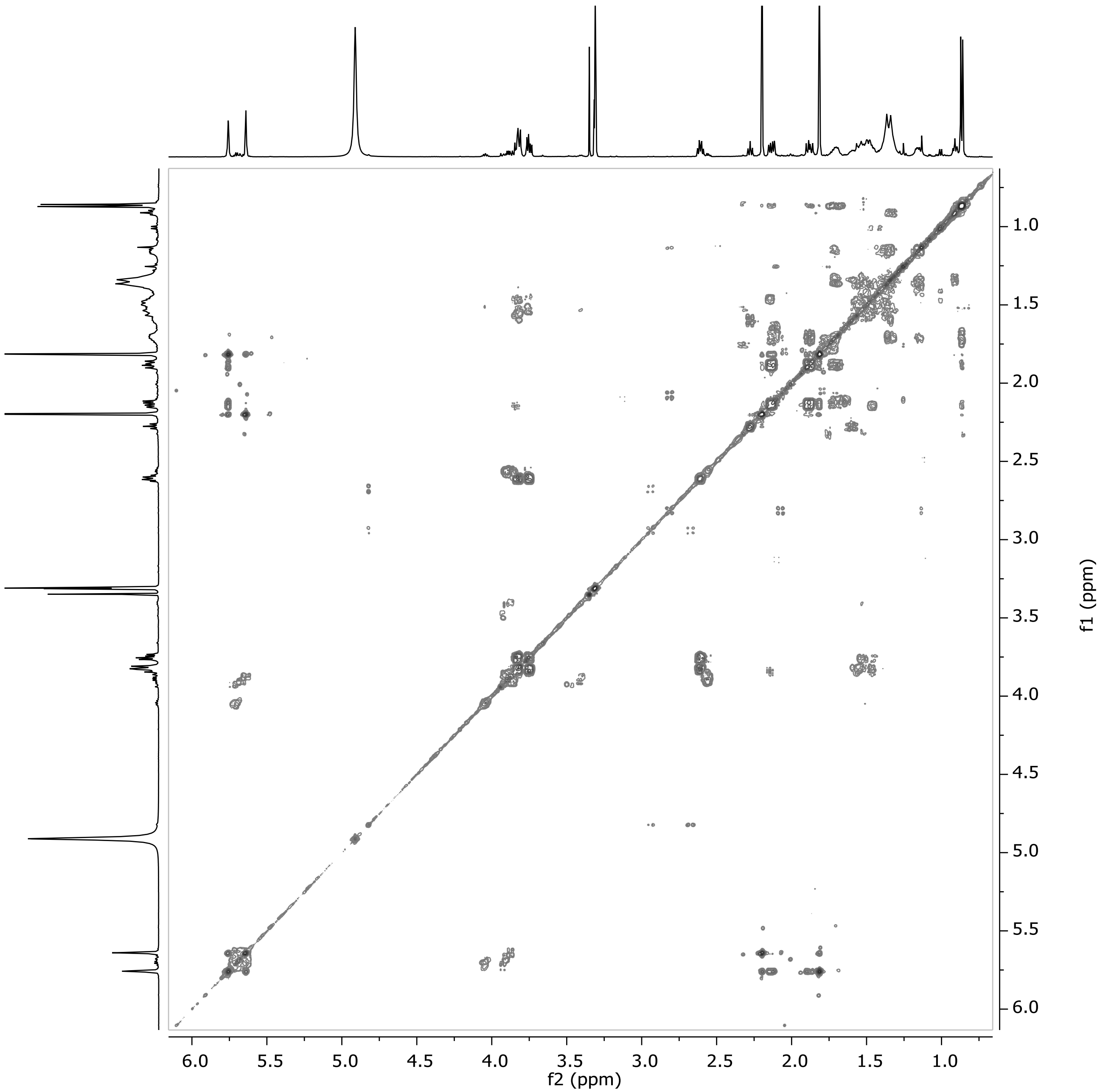

Figure S24. ^{1}H-^{1}H COSY spectrum of **4** in methanol-d_4 at 500 MHz.

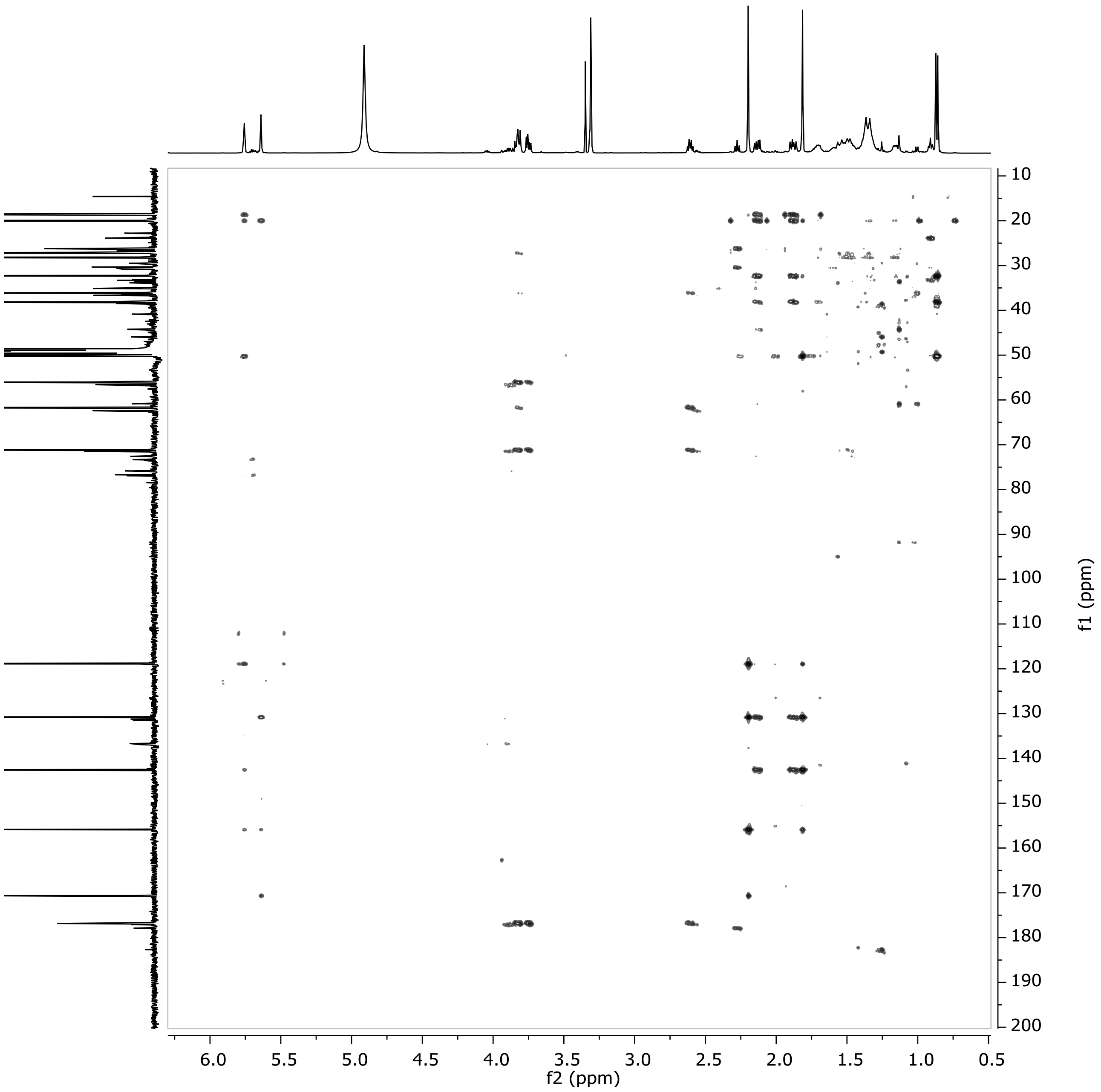

Figure S25. HMBC spectrum of **4** in methanol-d_4 at 500 MHz.

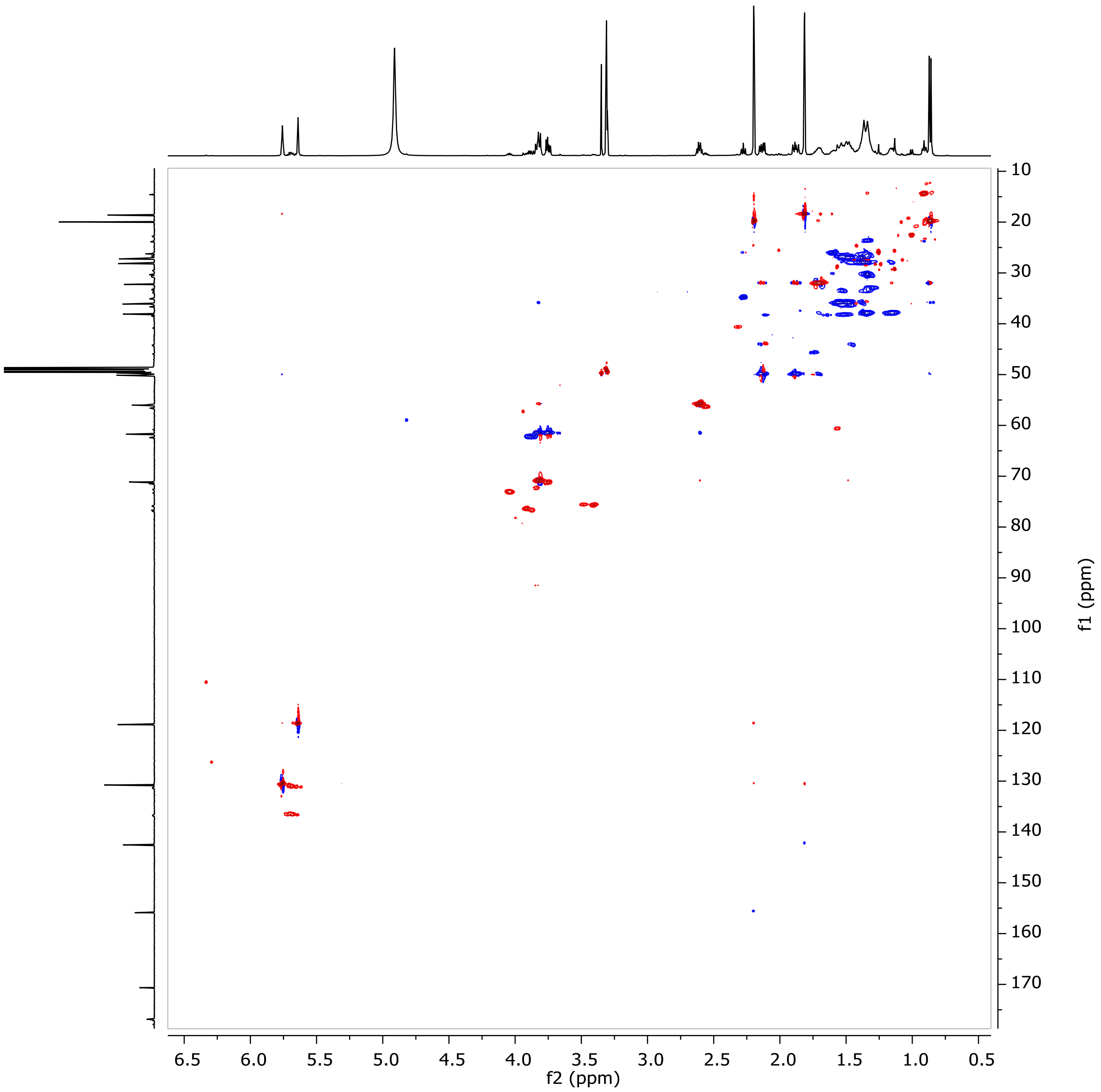

Figure S26. HSQC spectrum of **4** in methanol-d_4 at 500 MHz.

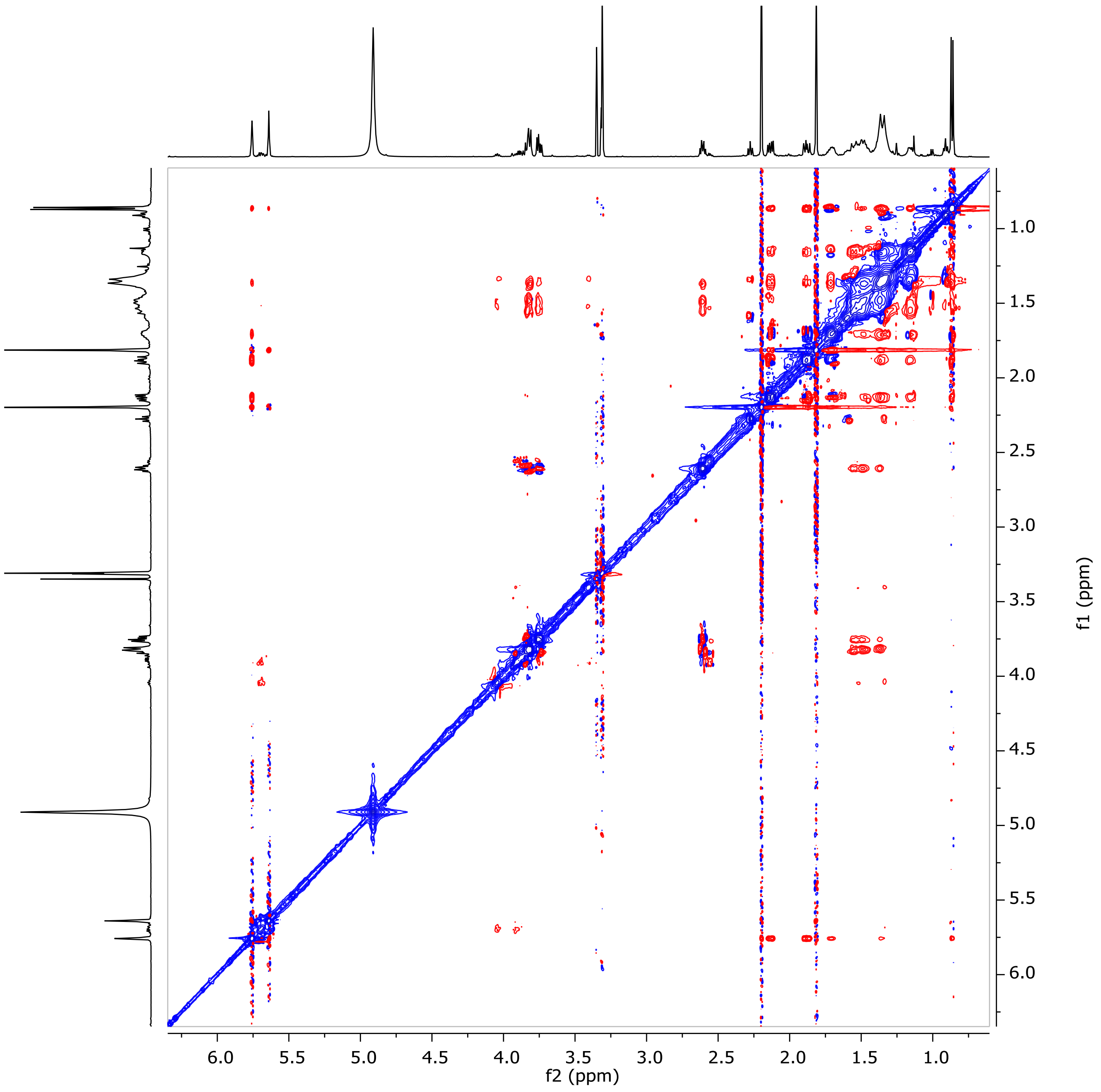

Figure S27. ROESY spectrum of **4** in methanol-d_4 at 500 MHz.

Generic Display Report

Analysis Info

Analysis Name	S:\PEOPLE\sel22_Sherif Elsayed\Phellinus\IHI 760\AmaZon\IHI 760 R2F8_RC8_01_51152.d	Acquisition Date	25.09.2023 23:43:43
Method	51152.m	Operator	tti
Sample Name	IHI 760 R2F8	Instrument	amaZon speed
Comment			

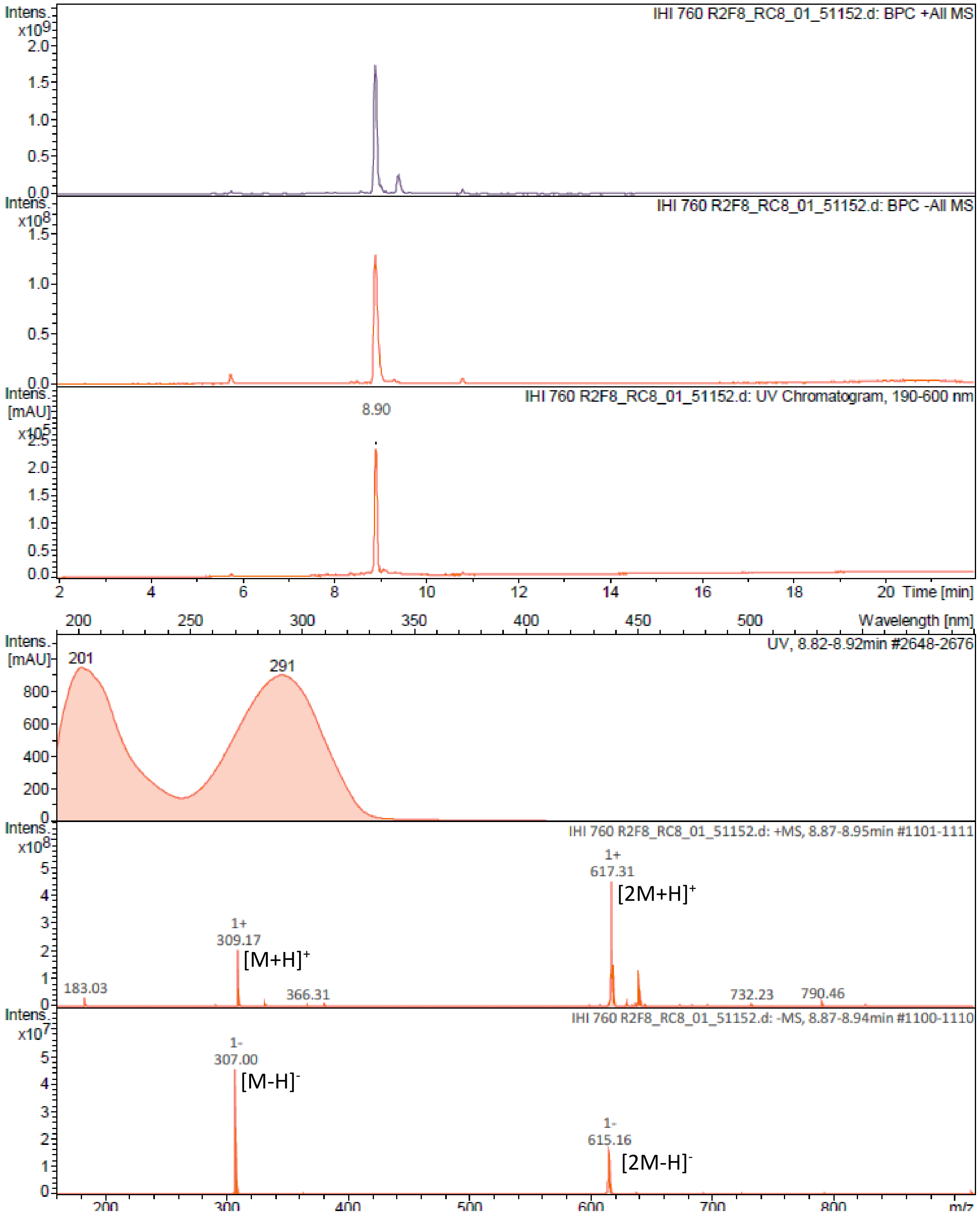

Bruker Compass DataAnalysis 4.4 printed: 19.10.2023 15:33:18 by: sel22 Page 1 of 1

Figure S28. LR-ESI-MS of **5**.

Generic Display Report

Analysis Info

		Acquisition Date	29.09.2023 13:56:31
Analysis Name	S:\PEOPLE\sel22_Sherif Elsayed\Phellinus\IHI 760\MaXis\IHI 760 R2F8_14_01_13359.d		
Method	pos_säure_10000_screening_ms_100_2500_line.m	Operator	ate06
Sample Name	IH 760 R2F8	Instrument	maXis
Comment	Screening01<br>Waters Acquity UPLC BEH C_18 1,7um 2.1x50mm		

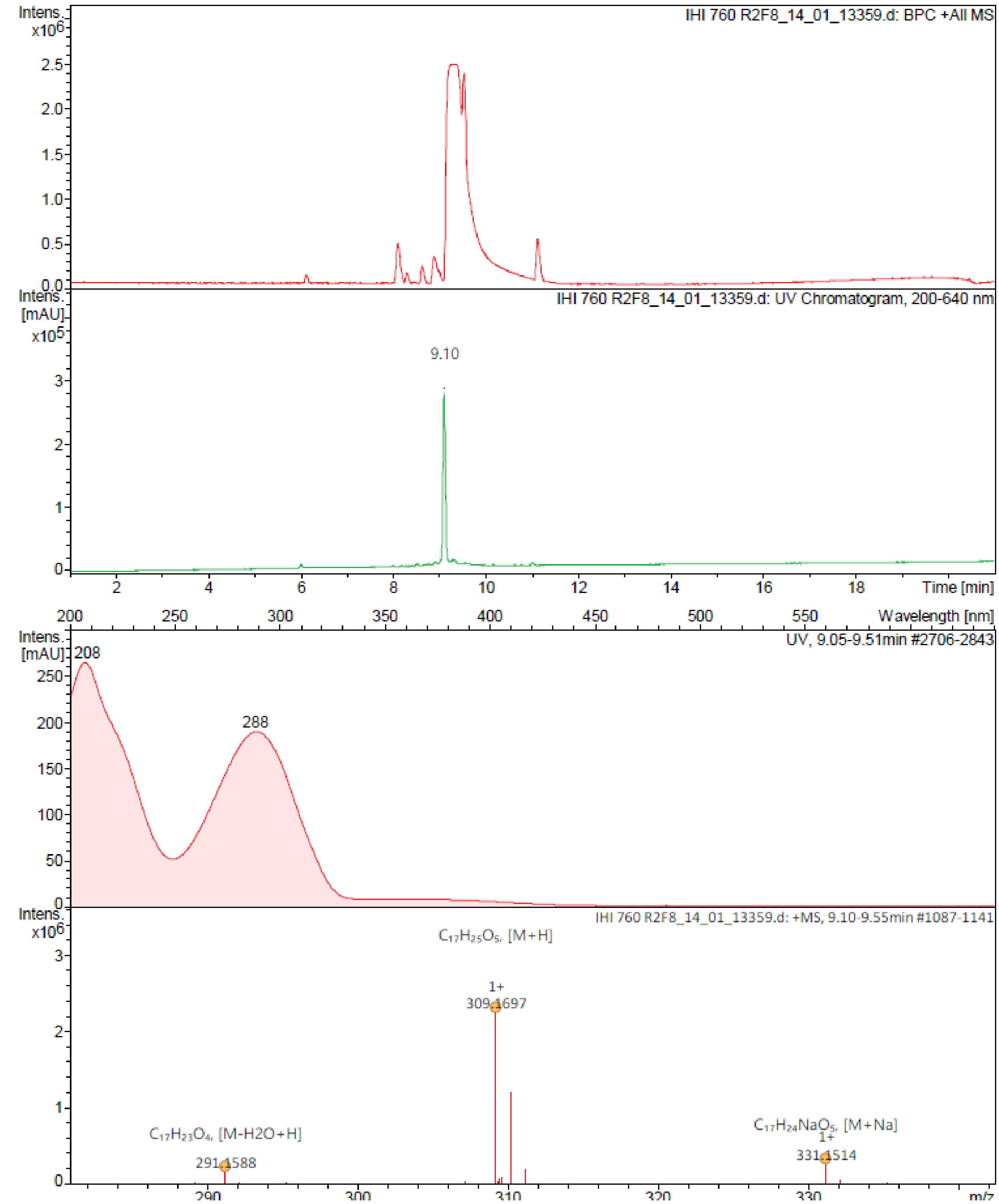

Bruker Compass DataAnalysis 4.4 printed: 19.10.2023 18:15:59 by: sel22 Page 1 of 1

Figure S29. HR-ESI-MS of **5**.

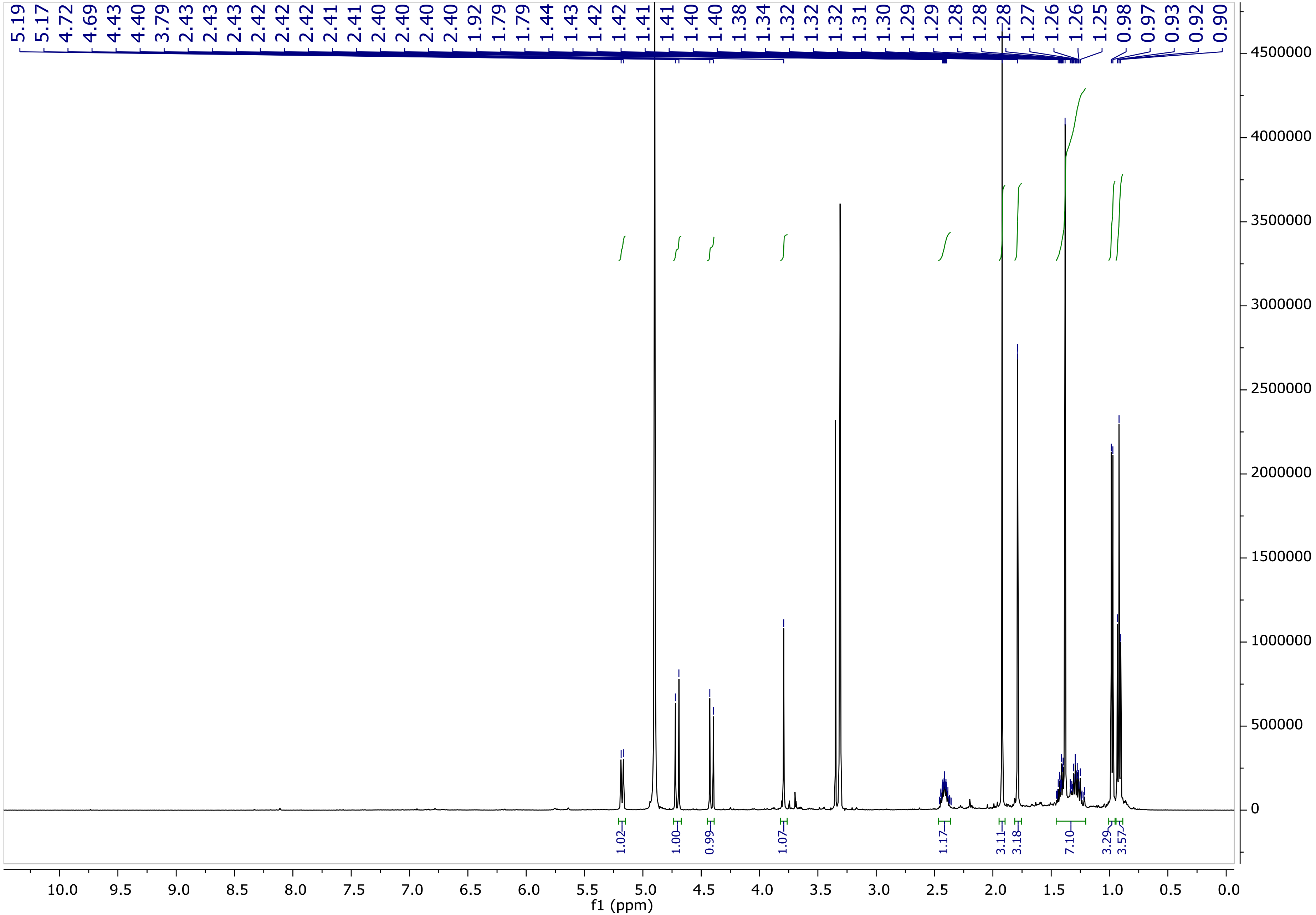

Figure S30. ^{1}H NMR spectrum of **5** in methanol-d_4 at 500 MHz.

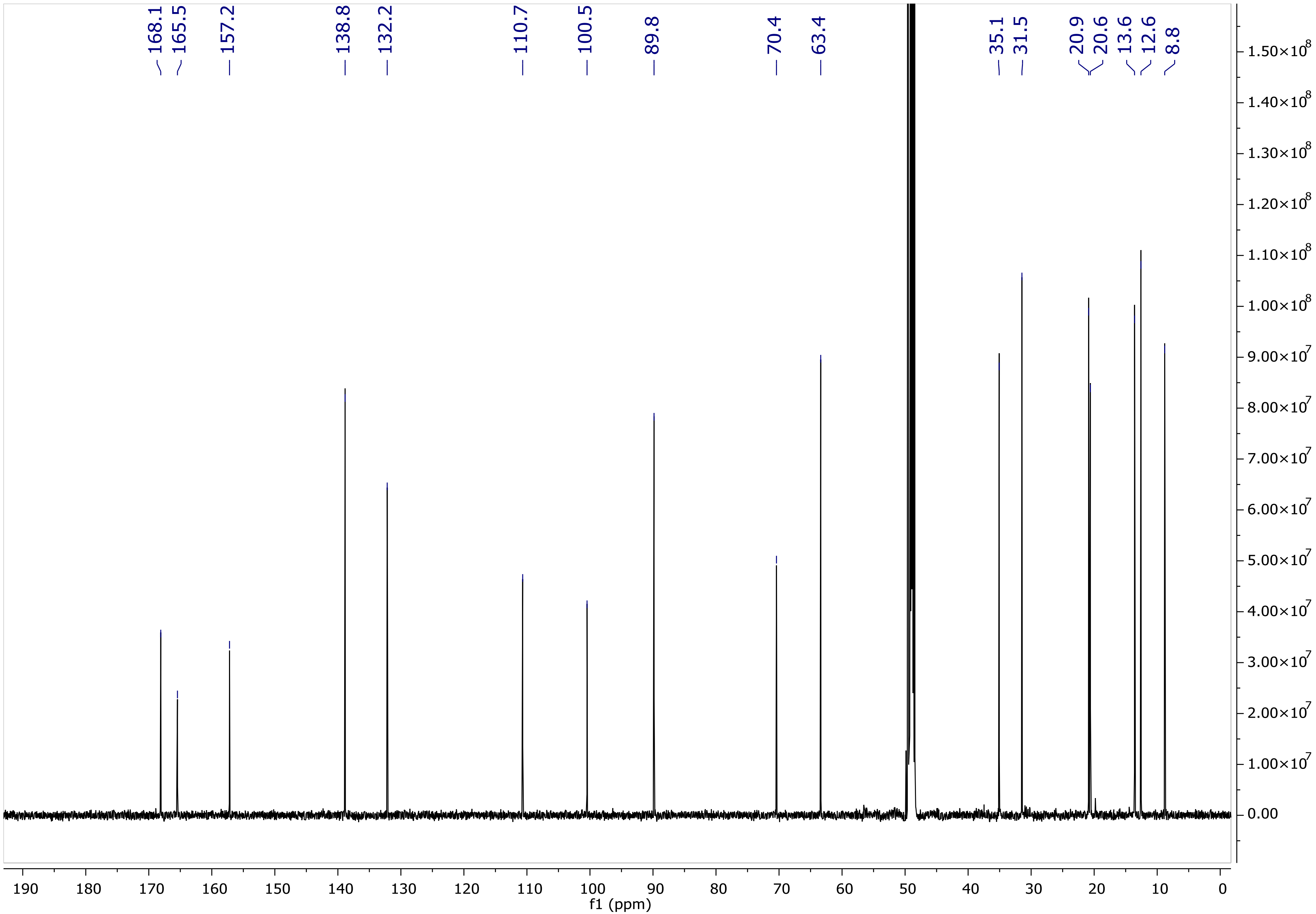

Figure S31. ^{13}C NMR spectrum of **5** in methanol-d_4 at 125 MHz.

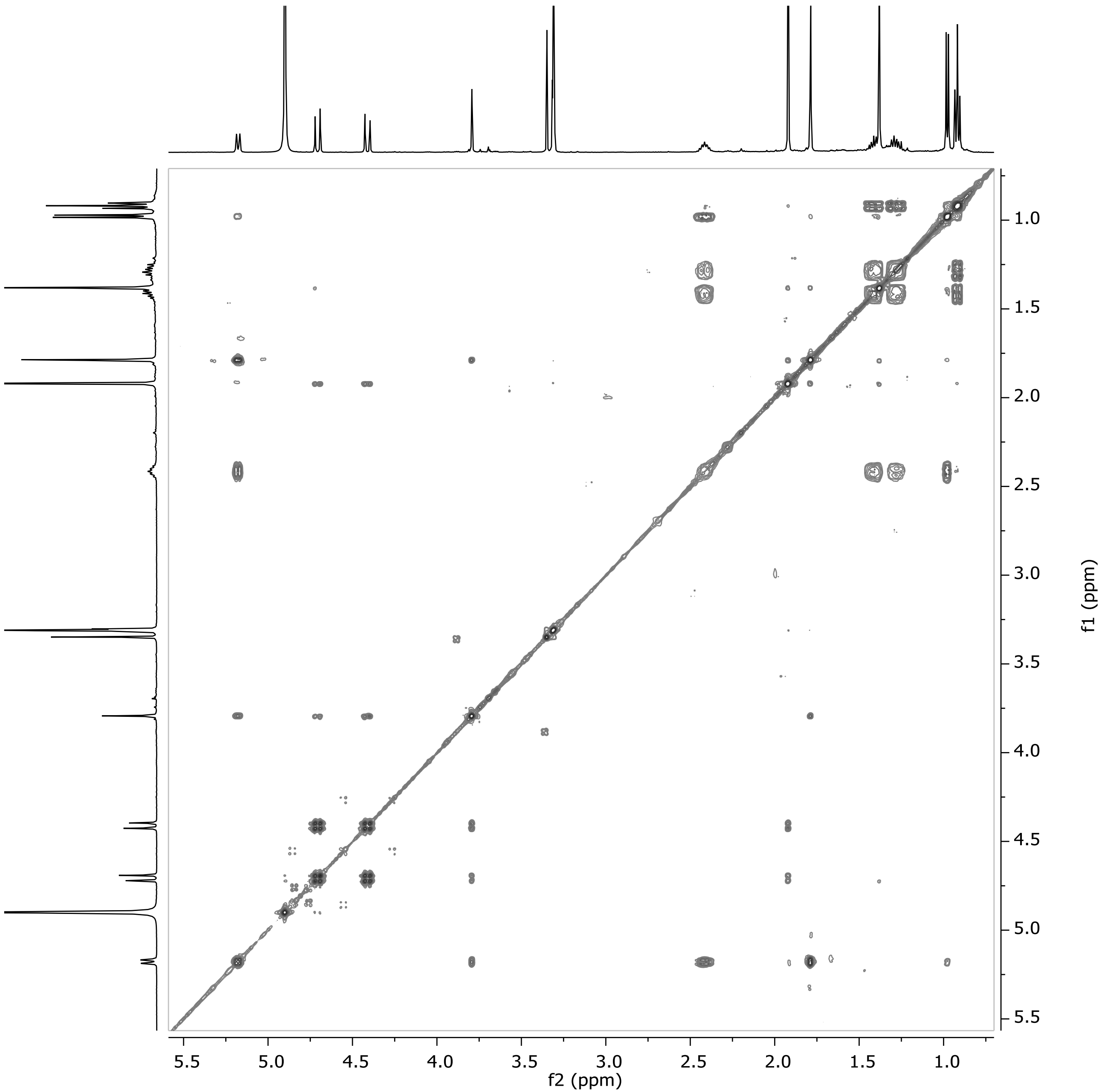

Figure S32. ^{1}H-^{1}H COSY spectrum of **5** in methanol-d_4 at 500 MHz.

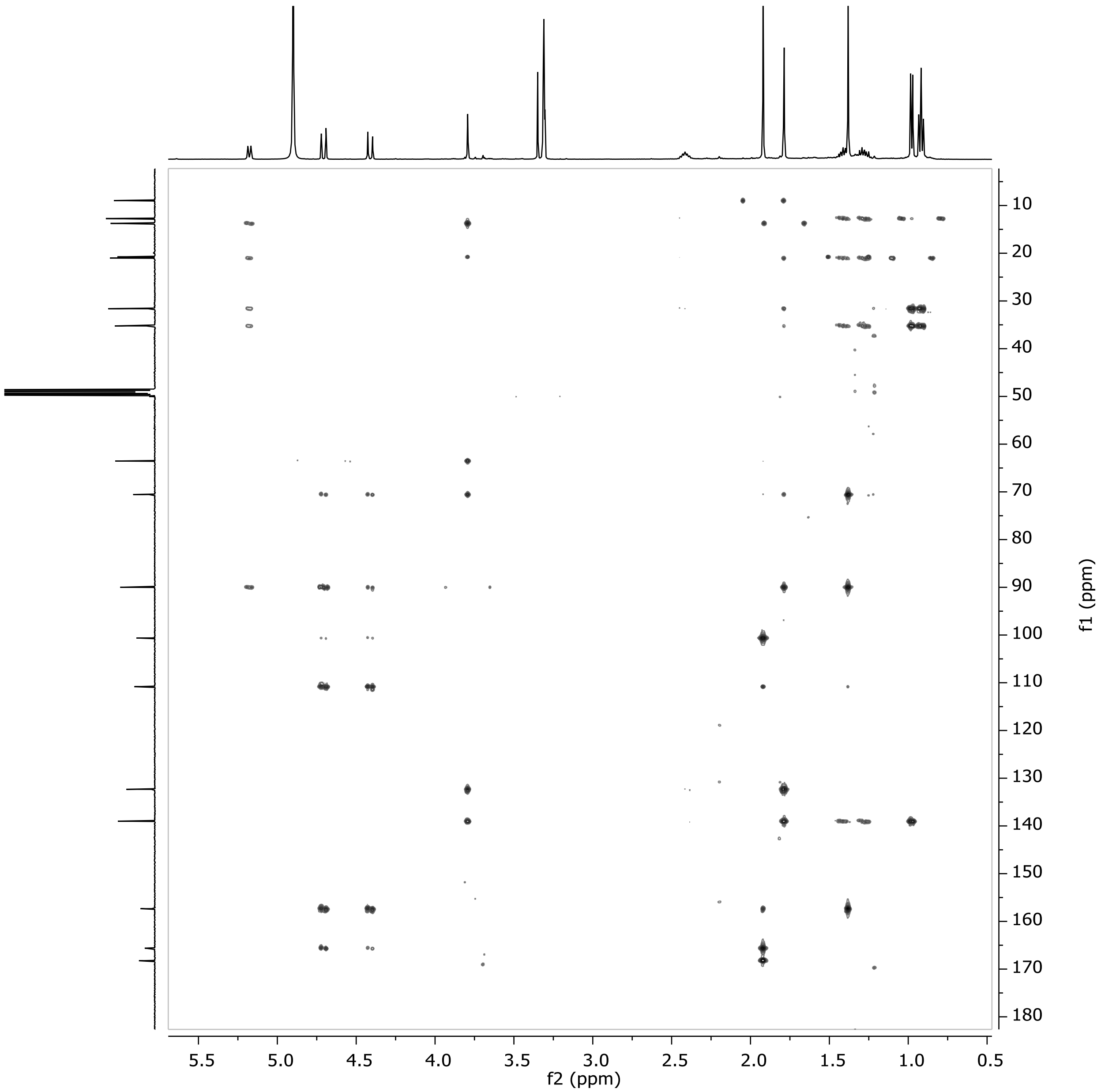

Figure S33. HMBC spectrum of **5** in methanol-d_4 at 500 MHz.

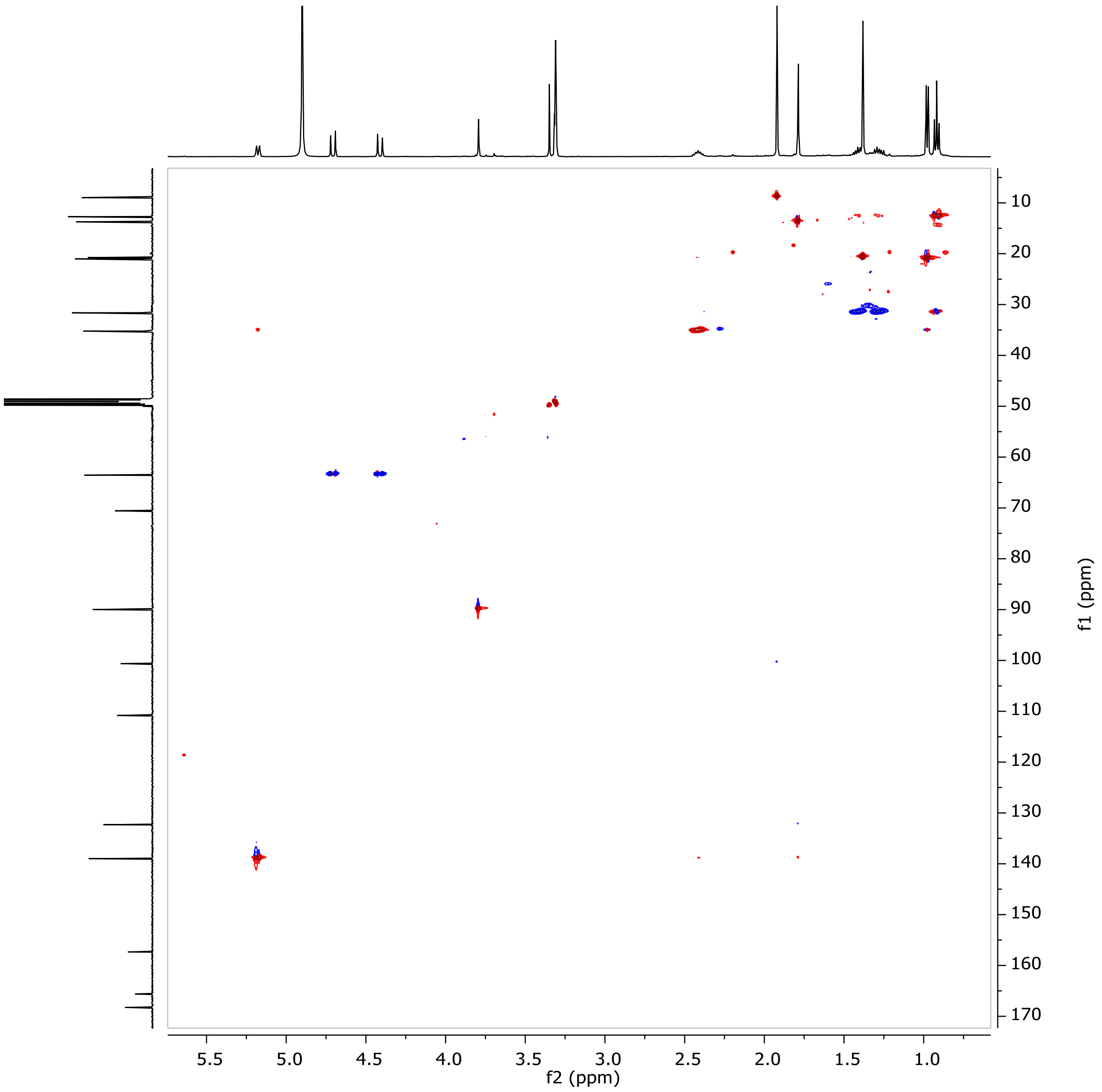

Figure S34. HSQC spectrum of **5** in methanol-d_4 at 500 MHz.

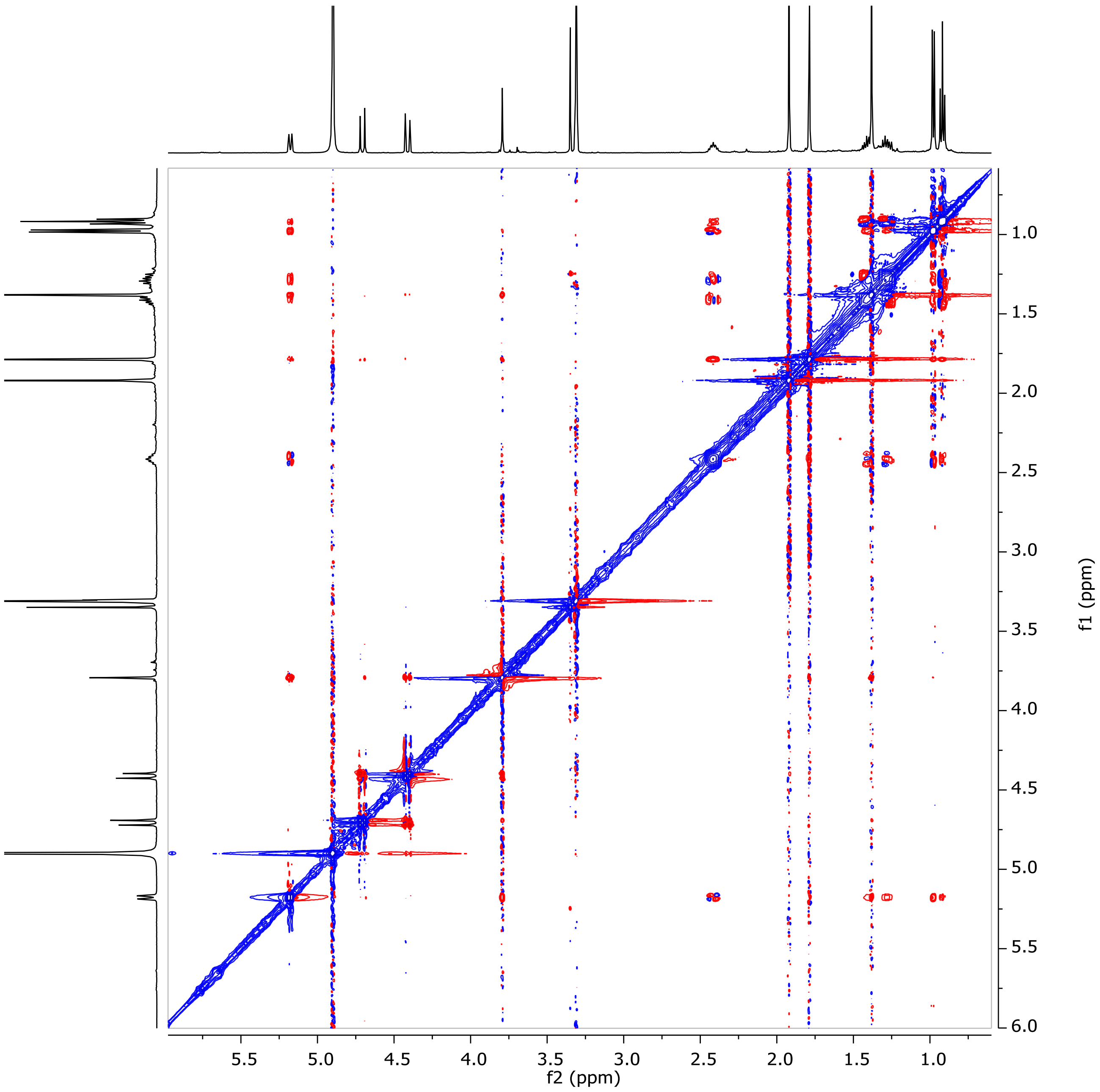

Figure S35. ROESY spectrum of **5** in methanol-d_4 at 500 MHz.

Display Report

Analysis Info

		Acquisition Date	21.09.2023 08:36:36
Analysis Name	S:\DATA\AmaZon\wsu20_Winnier Sum Chemutai\IHI CRUDE\IHI 760 Compounds\IHI 760 R1F2_RC2_01_50979.d		
Method	50979.m	Operator	tti
Sample Name	IHI 760 R1F2	Instrument	amaZon speed
Comment			

Acquisition Parameter

Ion Source Type	ESI	Ion Polarity	Negative	Alternating Ion Polarity	on
Mass Range Mode	UltraScan	Scan Begin	100 m/z	Scan End	2000 m/z
Accumulation Time	3481 µs	RF Level	100 %	Trap Drive	78.0
SPS Target Mass	1000 m/z	Averages	6 Spectra		

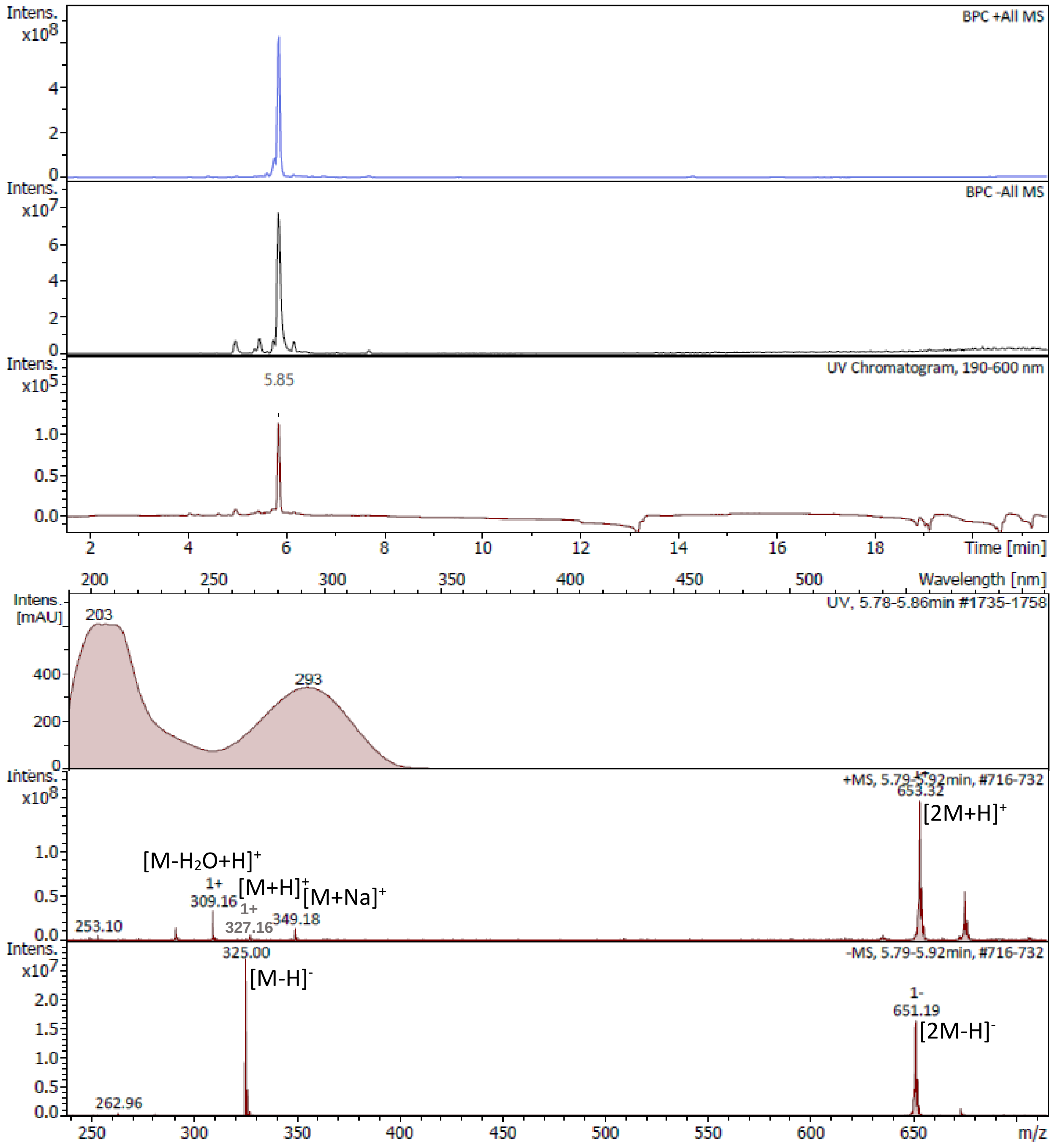

Figure 36. LR-ESI-MS of **6**/**7**.

Generic Display Report

Analysis Info

Analysis Name	S:\PEOPLE\sel22_Sherif Elsayed\Phellinus\IHI 760\MaXis\IHI 760 R2F3_11_01_13356.d	Acquisition Date	29.09.2023 12:23:42
Method	pos_säure_10000_screening_ms_100_2500_line.m	Operator	ate06
Sample Name	IH 760 R2F3	Instrument	maXis
Comment	Screening01<br>Waters Acquity UPLC BEH C_18 1,7um 2.1x50mm		

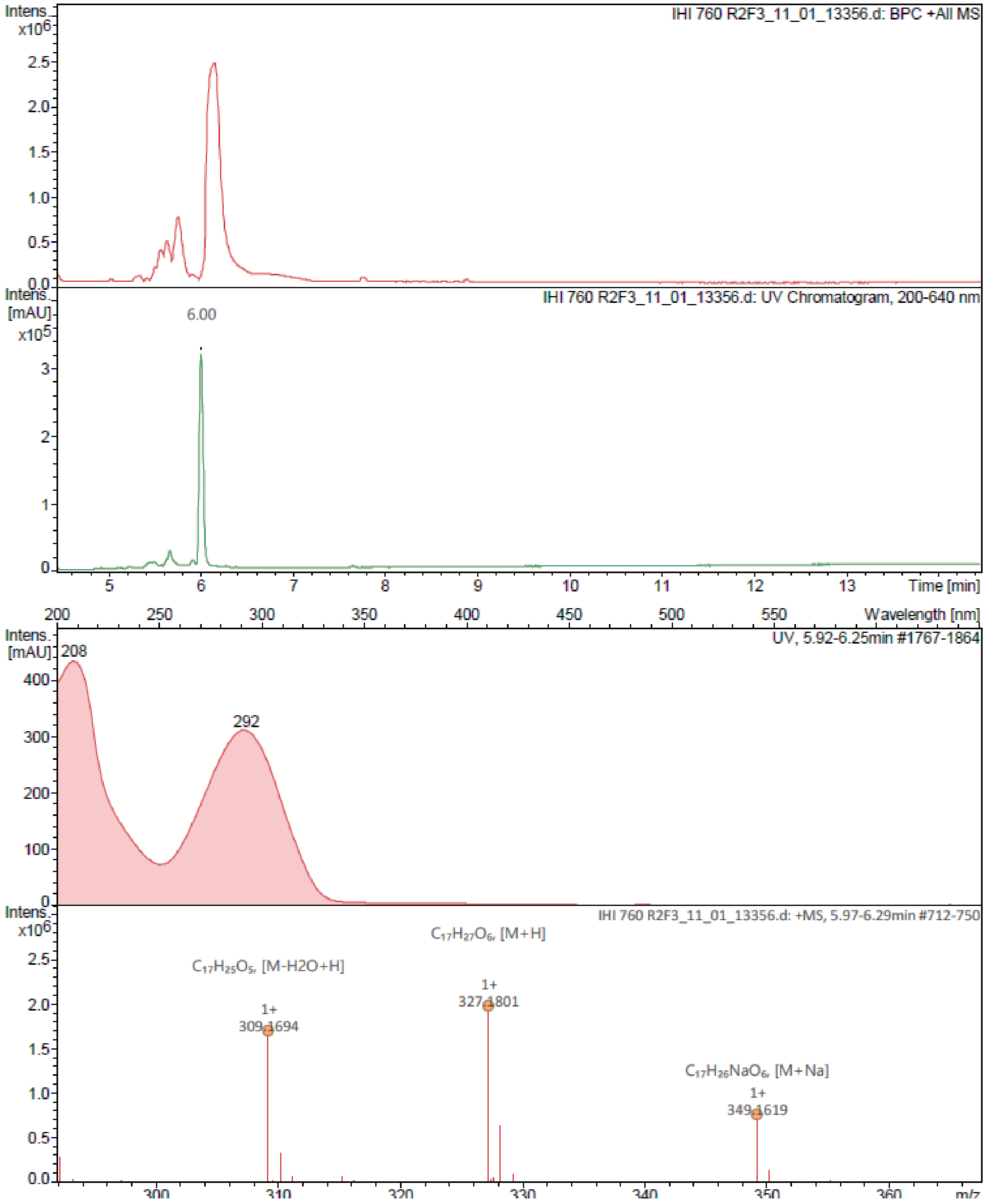

Bruker Compass DataAnalysis 4.4 printed: 19.10.2023 17:51:27 by: sel22 Page 1 of 1

Figure S37. HR-ESI-MS of **6**/**7**.

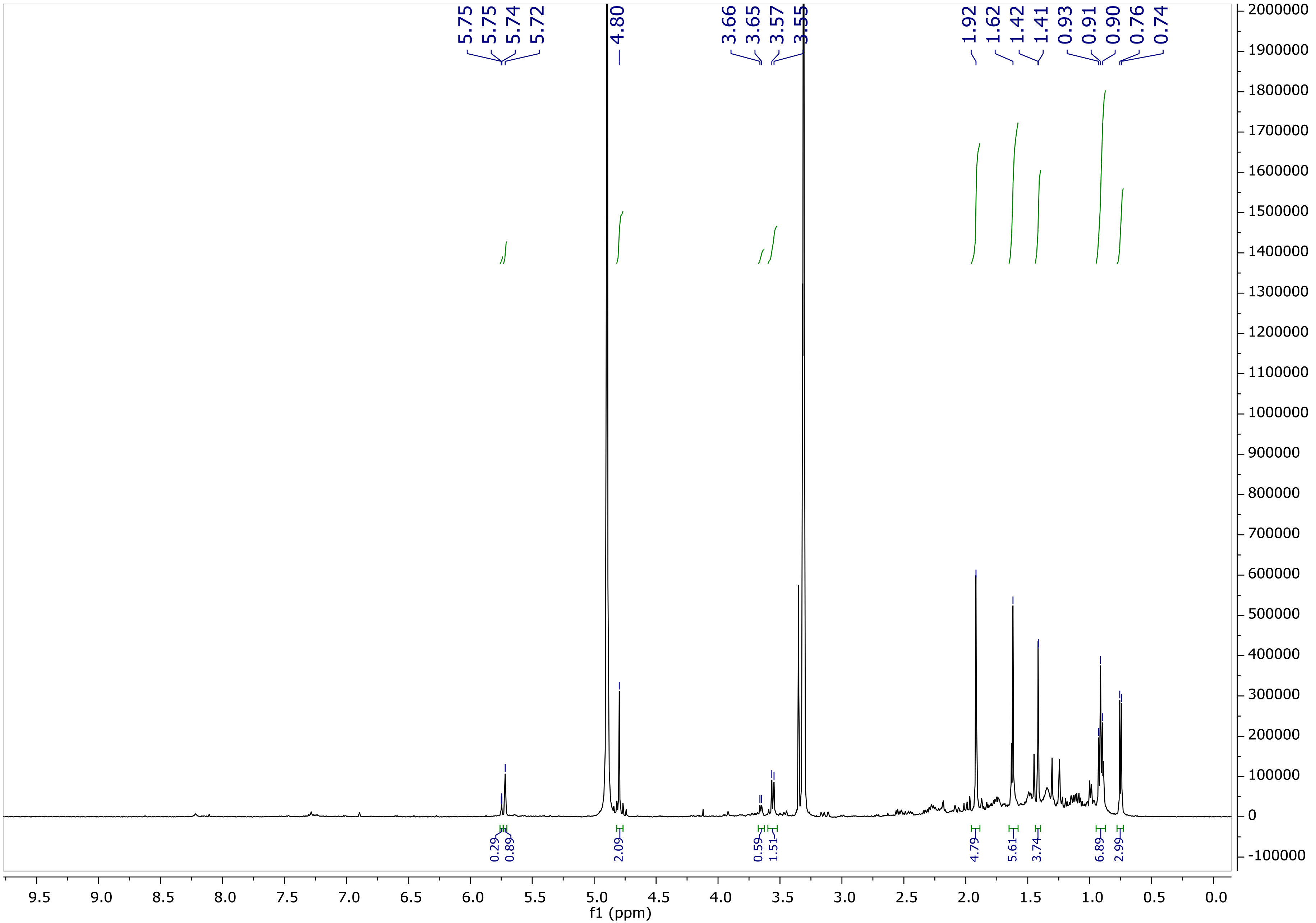

Figure S38. ^{1}H NMR spectrum of **6** and **7** (1:3) in methanol-d_4 at 500 MHz.

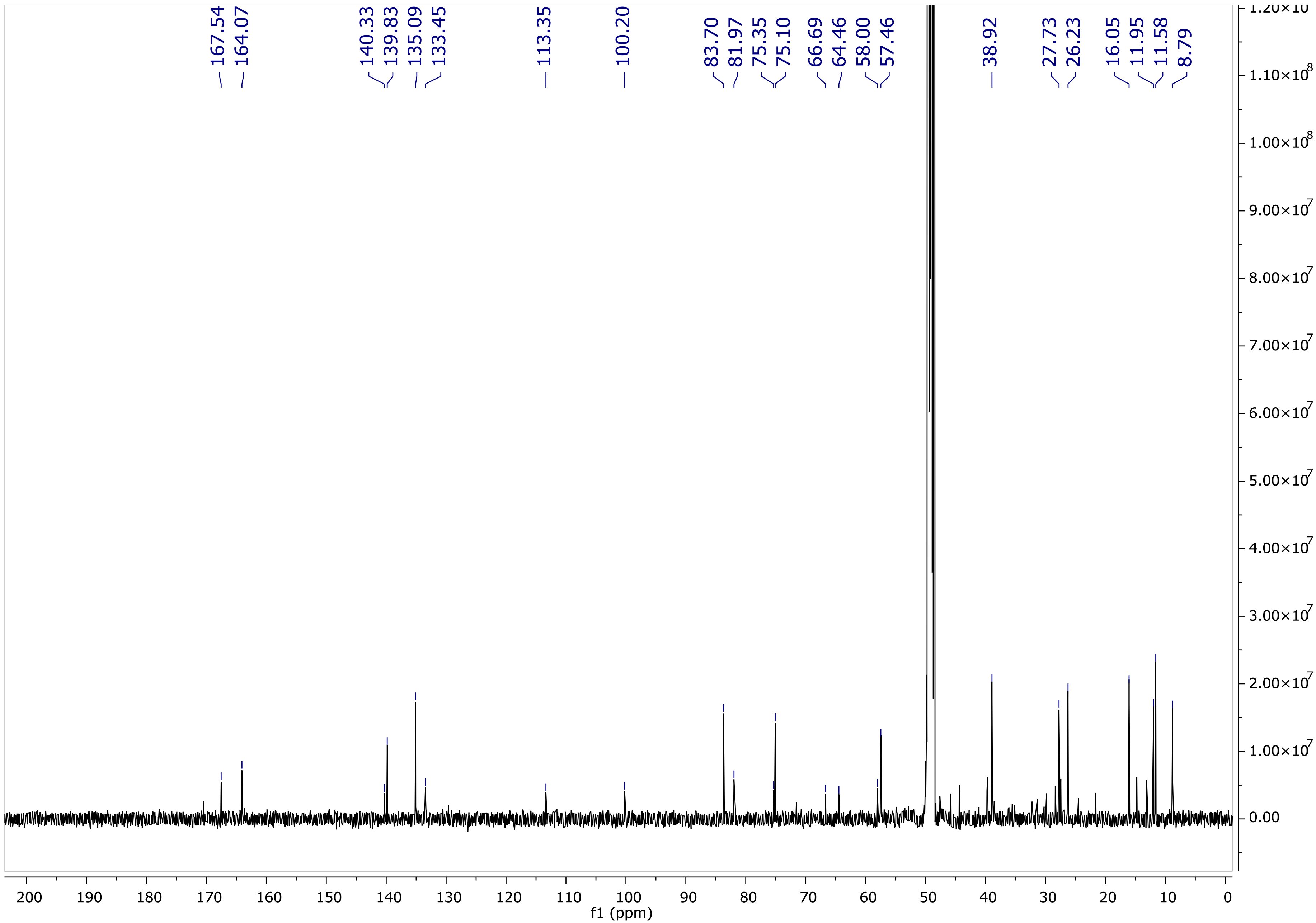

Figure S39. ^{13}C NMR spectrum of **6** and **7** (1:3) in methanol-d_4 at 125 MHz.

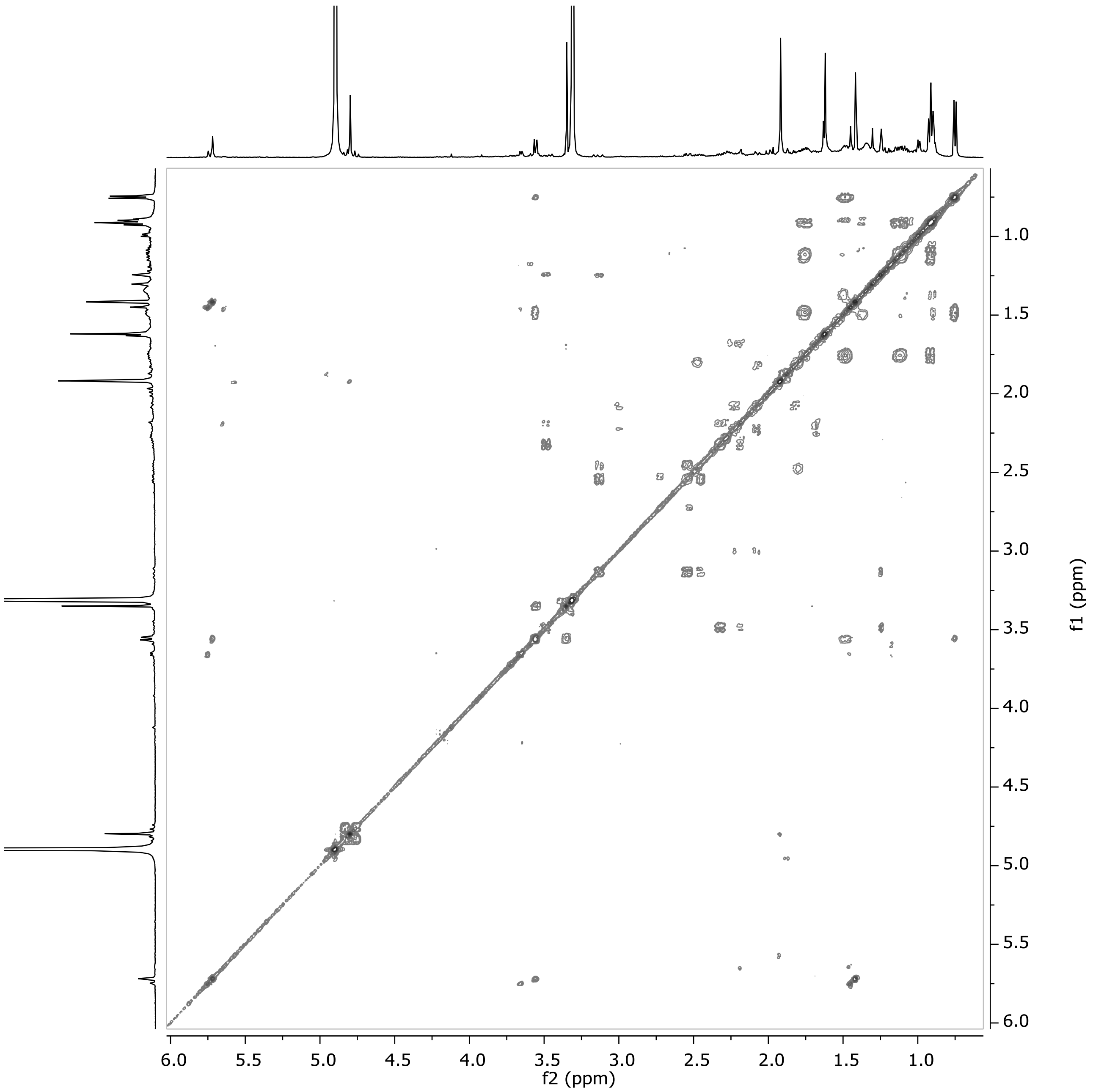

Figure S40. ^{1}H-^{1}H COSY spectrum of **6** and **7** (1:3) in methanol-d_4 at 500 MHz.

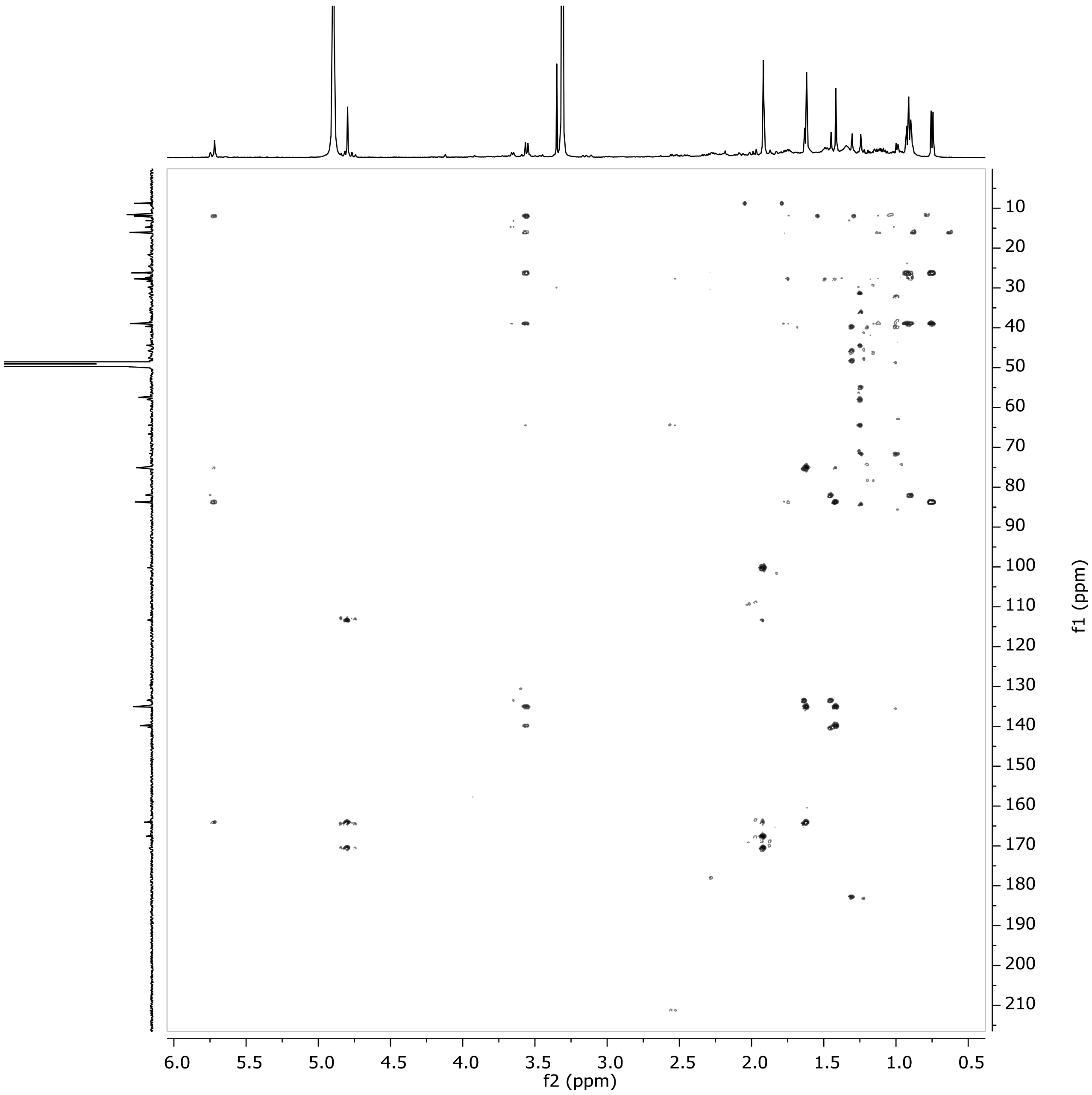

Figure S41. HMBC spectrum of **6** and **7** (1:3) in methanol-d_4 at 500 MHz.

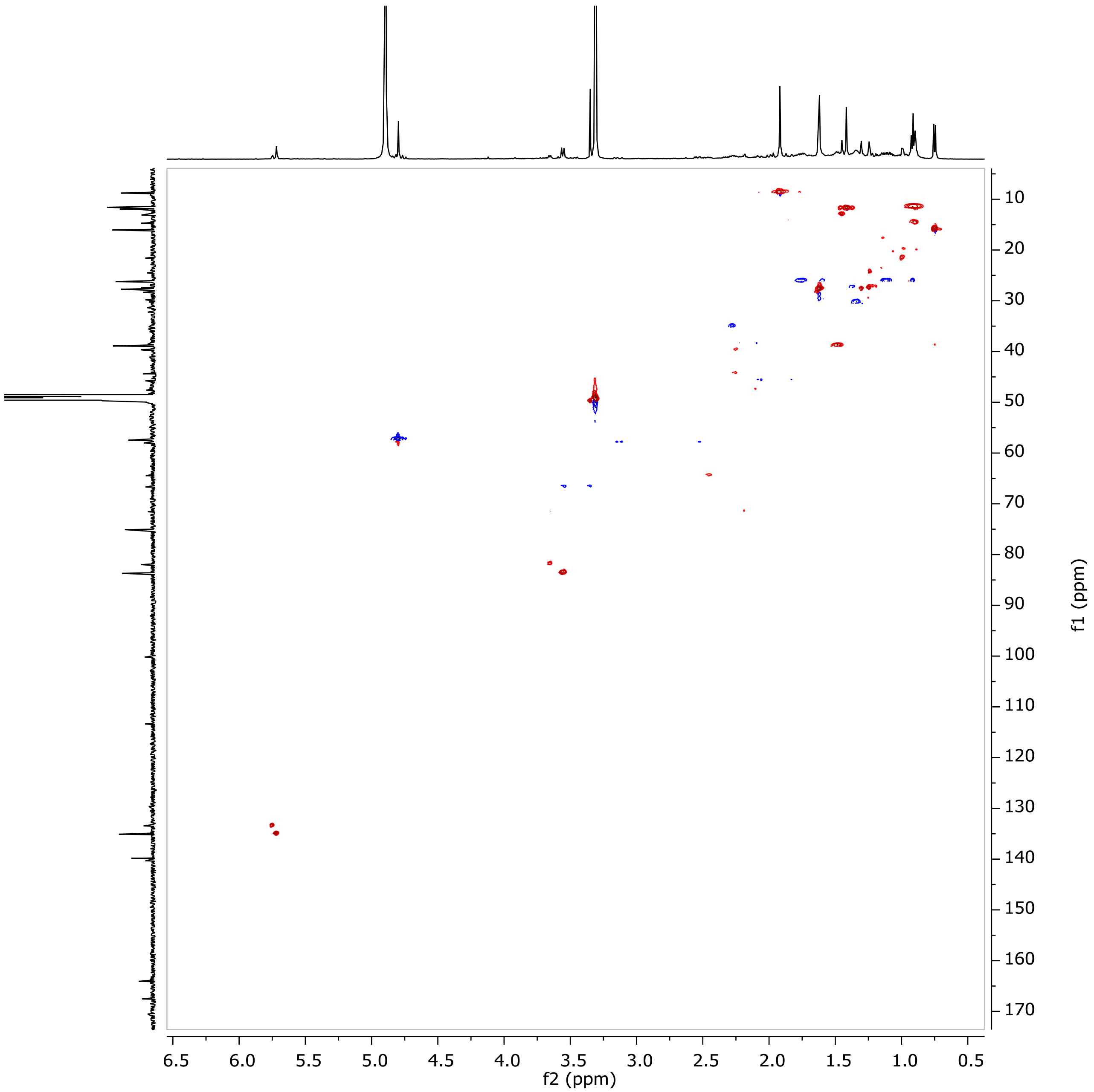

Figure S42. HSQC spectrum of **6** and **7** (1:3) in methanol-d_4 at 500 MHz.

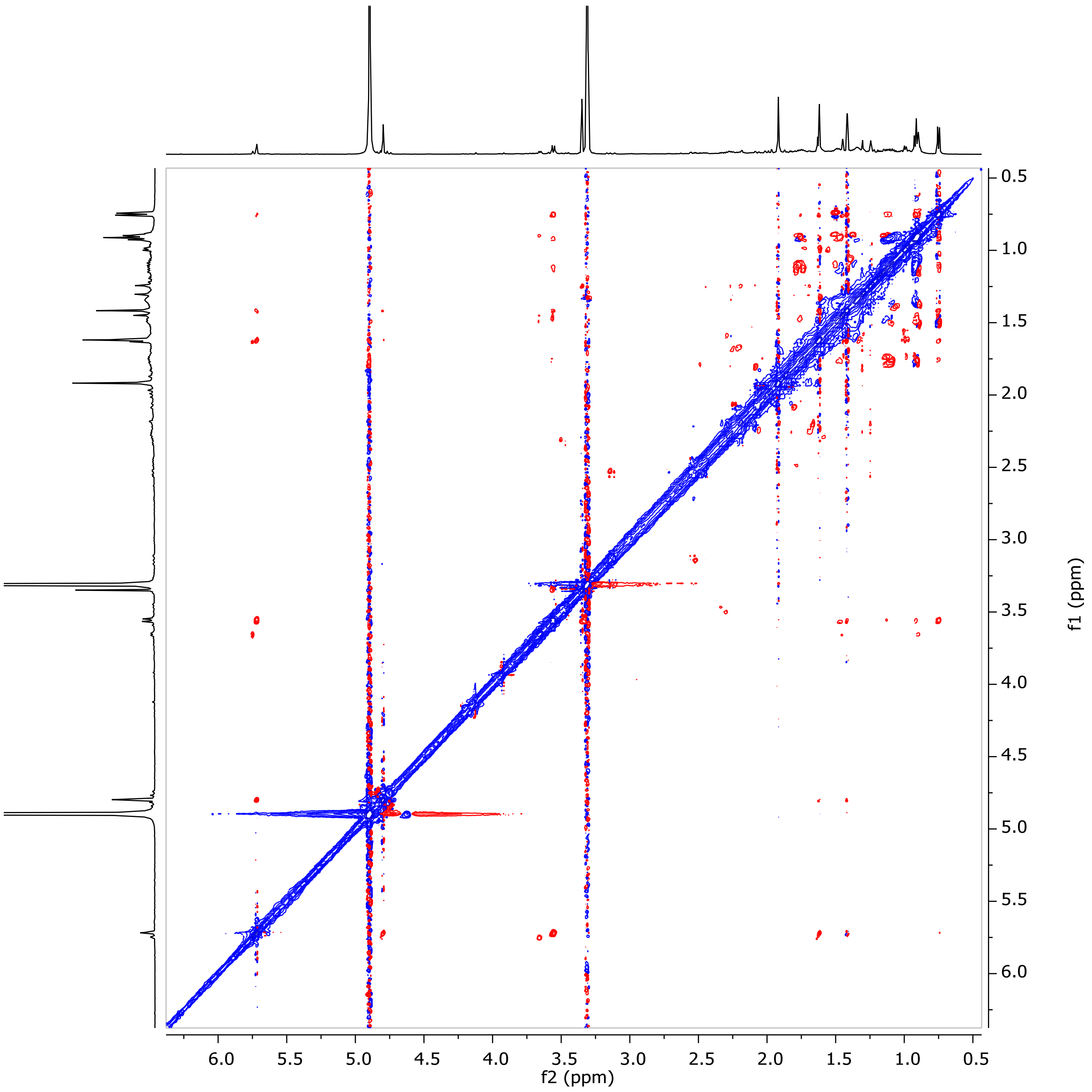

Figure S43. ROESY spectrum of **6** and **7** (1:3) in methanol-d_4 at 500 MHz.
